# The σ^E^ stress response is required for stress-induced mutation and amplification in *Escherichia coli*

**DOI:** 10.1111/j.1365-2958.2010.07213.x

**Published:** 2010-06-07

**Authors:** Janet L Gibson, Mary-Jane Lombardo, Philip C Thornton, Kenneth H Hu, Rodrigo S Galhardo, Bernadette Beadle, Anand Habib, Daniel B Magner, Laura S Frost, Christophe Herman, P J Hastings, Susan M Rosenberg

**Affiliations:** 1 Department of Molecular and Human GeneticsHouston, TX 77030-3411, USA; 2 Department of Biological Sciences, University of AlbertaEdmonton, Alberta T6G 2E9, Canada; 3 Department of Molecular Virology and MicrobiologyHouston, TX 77030-3411, USA; 4 Department of Biochemistry and Molecular BiologyHouston, TX 77030-3411, USA; 5 The Dan L Duncan Cancer Center, Baylor College of MedicineHouston, TX 77030-3411, USA

## Abstract

Pathways of mutagenesis are induced in microbes under adverse conditions controlled by stress responses. Control of mutagenesis by stress responses may accelerate evolution specifically when cells are maladapted to their environments, i.e. are stressed. Stress-induced mutagenesis in the *Escherichia coli* Lac assay occurs either by ‘point’ mutation or gene amplification. Point mutagenesis is associated with DNA double-strand-break (DSB) repair and requires DinB error-prone DNA polymerase and the SOS DNA-damage- and RpoS general-stress responses. We report that the RpoE envelope-protein-stress response is also required. In a screen for mutagenesis-defective mutants, we isolated a transposon insertion in the *rpoE* P2 promoter. The insertion prevents *rpoE* induction during stress, but leaves constitutive expression intact, and allows cell viability. *rpoE* insertion and suppressed null mutants display reduced point mutagenesis and maintenance of amplified DNA. Furthermore, σ^E^ acts independently of stress responses previously implicated: SOS/DinB and RpoS, and of σ^32^, which was postulated to affect mutagenesis. I-SceI-induced DSBs alleviated much of the *rpoE* phenotype, implying that σ^E^ promoted DSB formation. Thus, a third stress response and stress input regulate DSB-repair-associated stress-induced mutagenesis. This provides the first report of mutagenesis promoted by σ^E^, and implies that extracytoplasmic stressors may affect genome integrity and, potentially, the ability to evolve.

## Introduction

Stress-induced mutagenesis is a collection of mechanisms observed in bacterial, yeast and human cells in which mutagenesis pathways are activated in response to adverse conditions, such as starvation or antibiotic stresses (reviewed, Galhardo *et al*., 2007). These mechanisms enhance mutagenesis specifically during times of stress, and thus have the potential to increase genetic diversity upon which natural selection acts, potentially accelerating evolution, specifically when cells are maladapted to their environments, i.e. are stressed. These mechanisms are potentially important models for mutagenesis that drives antibiotic resistance (Cirz and Romesberg, 2007; Galhardo *et al*., 2007; Lopez and Blazquez, 2009; Petrosino *et al*., 2009; Kohanski *et al*., 2010) and pathogen–host evolutionary arms races (e.g. Prieto *et al*., 2006; Boles and Singh, 2008).

Although there appear to be multiple molecular mechanisms of stress-induced mutagenesis observed in different strains, organisms and environmental conditions, a strong common theme among them is the requirement for one or more cellular stress response(s). For example, induction of the SOS DNA-damage response is required for SOS ‘untargeted’ mutation of undamaged DNA (Witkin and Wermundsen, 1979), stress-induced reversion of a mutant *lac* gene in *Escherichia coli* cells starving on lactose medium (McKenzie *et al*., 2000), ciprofloxacin-induced resistance mutagenesis in *E. coli* (Cirz *et al*., 2005), bile-induced resistance mutagenesis in *Salmonella enterica* (Prieto *et al*., 2004; 2006;), and mutagenesis in aging *E. coli* colonies (Taddei *et al*., 1995). Similarly, the RpoS (σ^S^) general- or starvation-stress response is required for most of the pathways listed above, except ciprofloxacin-induced mutagenesis (Bjedov *et al*., 2003; Layton and Foster, 2003; Lombardo *et al*., 2004; J. Casadesus, pers. comm.) and additionally for stress-induced gene amplifications during starvation in *E. coli* (Lombardo *et al*., 2004), stress-induced excisions of coliphage Mu (Gomez-Gomez *et al*., 1997; Lamrani *et al*., 1999), and stress-induced point mutagenesis (Saumaa *et al*., 2002) and transposon movement (Ilves *et al*., 2001) in *Pseudomonas putida*. The stringent response to amino acid starvation in *E. coli* (Wright *et al*., 1999) and *Bacillus subtilis* (Rudner *et al*., 1999b), and the ComK competence response to starvation in *B. subtilis* (Sung and Yasbin, 2002), are required for mutation pathways induced by starvation stresses. In human cells, two different stress responses to hypoxia provoke two separate mechanisms of genome instability (Bindra *et al*., 2007; Huang *et al*., 2007). Coupling of mutation pathways to cellular stress responses appears to be how mutagenesis is targeted specifically to times of stress. The stress responses are thus the cornerstone of the regulation of these mutation pathways, and their identities provide windows into the biological/environmental stressors that elicit the mutagenesis responses.

In the *E. coli* Lac assay for starvation-stress-induced mutagenesis (Cairns and Foster, 1991), either reversion of a frameshift mutation by compensatory frameshift mutation (Foster and Trimarchi, 1994; Rosenberg *et al*., 1994) in an F′-borne *lac* gene, or amplification of the leaky *lac* allele to multiple copies (Hastings *et al*., 2000; Powell and Wartell, 2001), allows growth of cells starving on lactose medium. The frameshift (‘point’ mutation) pathway requires double-strand-break (DSB)-repair proteins (Harris *et al*., 1994; 1996; Foster *et al*., 1996), TraI, an F-encoded single-strand endonuclease or an I-SceI-generated DSB (Ponder *et al*., 2005), the DinB error-prone DNA polymerase (Foster, 2000; McKenzie *et al*., 2001), and two stress responses: the SOS DNA-damage response (McKenzie *et al*., 2000) and the general-stress response regulated by RpoS (Layton and Foster, 2003; Lombardo *et al*., 2004), both of which upregulate *dinB* transcription. Our lab has provided evidence that supports a mechanism in which the point mutations are formed in acts of error-prone DSB repair. The normally high-fidelity DSB-repair mechanism switches to an error-prone mutagenic pathway that uses DinB during the SOS and RpoS responses (Ponder *et al*., 2005). [Alternative models discussed by Roth *et al*. (2006) and Galhardo *et al*. (2007) and below]. The GroEL chaperone also modulates DinB activity (Layton and Foster, 2005). GroEL is an essential protein complex that is both constitutively expressed and upregulated during the RpoH/σ^32^-controlled cytoplasmic unfolded-protein-stress or heat-shock response, and also directly regulates the activity of σ^32^ (Guisbert *et al*., 2004).

The *rpoE* gene encodes the σ^E^ transcription factor, which positively regulates the envelope-stress response to extracytoplasmic unfolded proteins. Originally identified as a heat-shock factor, σ^E^ is essential for growth in *E. coli* at all temperatures although the exact nature of the σ^E^ requirement for growth is not known (Hiratsu *et al*., 1995; Rouviere *et al*., 1995; De Las Penas *et al*., 1997b). In *E. coli*, σ^E^ expression during either exponential or stationary phase up- or downregulates as many as 200 genes involved in all areas of metabolism (Kabir *et al*., 2004; Rhodius *et al*., 2006). Although many σ^E^-regulated genes encode proteins that promote either maintenance or synthesis of the cell envelope, a significant number encode cytoplasmic proteins that function in transcription, translation, and DNA synthesis and repair (Rhodius *et al*., 2006).

The regulation of σ^E^, and thus the stress response that it controls, is complex and entails at least two independent pathways (Ades, 2004). First, RseA, the σ^E^ anti-sigma factor, is an integral transmembrane protein that binds σ^E^, sequestering it at the cytoplasmic face of the inner membrane (Missiakas *et al*., 1997; De Las Penas *et al*., 1997a; Campbell *et al*., 2003). An inner membrane protease cascade (Ades, 2008), part of a regulatory pathway termed RIP (regulatory intramembrane proteolysis) conserved from bacteria to mammals (Rudner *et al*., 1999a; Brown *et al*., 2000), is responsible for degradation of RseA. Upon stress, unfolded outer membrane proteins trigger RseA proteolysis thereby releasing σ^E^ into the cytoplasm where it can associate with RNA polymerase and activate transcription of the σ^E^-regulon genes (Kanehara *et al*., 2003; Walsh *et al*., 2003). Turnover of RseA is continuous, but upon stress, degradation accelerates until the stress is removed, allowing increased σ^E^ levels in the cytoplasm and induction of transcription of the σ^E^ regulon (Ades *et al*., 1999; Alba *et al*., 2001; 2002; Kanehara *et al*., 2003; Grigorova *et al*., 2004).

In addition to activation by unfolded membrane proteins, σ^E^ is also regulated growth-phase-dependently by the stringent response, via a less well-defined pathway. This results in stationary-phase induction of σ^E^ in response to the alarmone, ppGpp (Costanzo and Ades, 2006; Costanzo *et al*., 2008).

In this study, we show that activation of the σ^E^ stress response is required for stress-induced mutagenesis in the *E. coli* Lac system. We describe a transposon insertion that disrupts σ^E^ expression during stress. We show that stress-inducible *rpoE* expression (the σ^E^ stress response) is not required for viability but the constitutive *rpoE* expression is, and we correlate the stress response, rather than the constitutive essential function of σ^E^, with formation of stress-induced point mutants and with maintenance (and thus accumulation of) DNA amplification. We show that σ^E^ plays at least two roles in the point-mutation pathway, one of them involving formation of DSBs. This adds a third stress response to those controlling stress-induced point mutagenesis, a second to that controlling amplification, and additionally adds genetic and genomic instability to the repertoire of consequences of the σ^E^ extracytoplasmic protein stress response in *E. coli*.

## Results

### *rpoE2072*::Tn*10*dCam is a non-null stress–response-defective mutation that allows cell viability without extragenic suppressor mutations

We developed a genetic screen, used it with transposon mutagenesis to identify mutants defective in stress-induced mutagenesis in the Lac assay (Experimental procedures in *Supporting information*), and identified a new mutation that affects the *rpoE* gene, designated *rpoE2072*::Tn*10*dCam. *rpoE2072*::Tn*10*dCam was moved by P1 transduction into a ‘clean’ (non-mutagenized) assay strain and characterized.

**Fig. 1:**
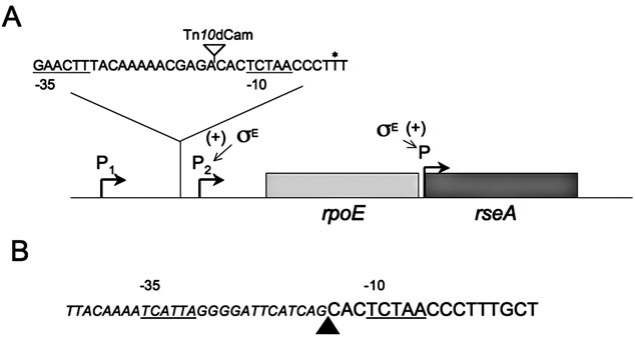
Location and consequences of a Tn*10*dCam insertion in the σ^E^-dependent *rpoE* P2 promoter. A. The Tn*10*dCam insertion site determined by sequence analysis, the transcription start site (*), and the –10 and –35 sequences (underlined) are indicated (Rouviere *et al*., 1995). P_1_ and P_2_ denote the two previously mapped σ^E^ promoters (Rouviere *et al*., 1995). Also shown is the location of the promoter between *rpoE* and *rseA* (Rhodius *et al*., 2006). (+) indicates positive transcriptional regulation by σ^E^. B. A putative –35 sequence (underlined) is supplied by the transposon. Location of the Tn*10*dCam insertion site is indicated by symbol (▴). Sequence to left of symbol (Italics) is Tn*10* sequence and to the right (large type) is 5′ sequence of *rpoE*.

We find that the *rpoE2072*::Tn*10*dCam insertion lies between the −10 and −35 regions of σ^E^-dependent (P2) gene-proximal promoter (Fig. 1A). Such an insertion might be expected to block transcription entirely and, given that *rpoE* is an essential gene (De Las Penas *et al*., 1997b; Alba and Gross, 2004), allow growth only of isolates with additional suppressor mutations that restore viability. *rpoE* null mutants acquire unlinked suppressor mutations (De Las Penas *et al*., 1997b). Using co-transduction experiments, we show that *rpoE2072*::Tn*10*dCam insertion mutant does not contain or require unlinked suppressor mutations for its viability (Fig. S1). We used a kanamycin-resistance marker in *yfhH*, a non-essential gene partially linked with *rpoE2072*::Tn*10*dCam, to transduce cells carrying neither the *rpoE* nor *yfhH*::Kan mutation with phage grown on the *yfhH*::Kan *rpoE2072*::Tn*10*dCam donor (Fig. S1). Co-transductant frequencies of 51 ± 4% Cam^R^/Kan^R^ transductants, and 67 ± 2% Kan^R^/Cam^R^ transductants (Fig. S1) show that although there is a slight bias against acquisition of the *rpoE2072* allele, the *rpoE2072*::Tn*10*dCam insertion does not induce lethality when transduced into a clean (suppressor-mutation-free) strain background (Fig. S1). Thus unlinked suppressor mutations are not required for the mutant's growth. Although *rpoE2072*::Tn*10*dCam is not a null mutation (Fig. S1) this allele causes a slight temperature-sensitive phenotype with similar numbers of cfu at 30°C and 37°C, but about twofold fewer at 42°C relative to 30°C (not shown).

Two likely explanations for the viability *of rpoE2072* cells are either that the 3′ end of the transposon supplies a poorly conserved −35 sequence (Fig. 1B) or that transcription is being initiated from an outward-reading promoter within the transposon allowing some σ^E^ expression. Data below indicate that the insertion affects the expression from the σ^E^-dependent P2 promoter.

**Fig. 2:**
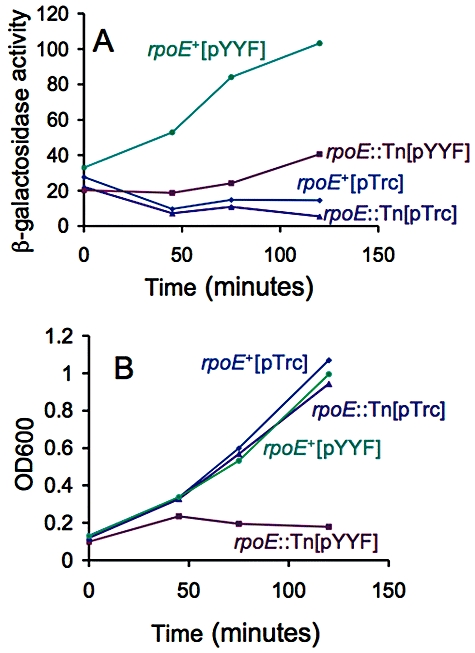
Stress induction of a σ^E^-regulated promoter is inhibited in *rpoE2072*::Tn*10*dCam cells. β-Galactosidase activity expressed from the *rpoH* P3 promoter was measured following induction of the OMP C-terminal peptide, YYF, from pBA166, or with the control vector plasmid pTrc99. The experiment was repeated twice with similar results. A. β-Galactosidase activity plotted over time following IPTG addition to LBH cultures. Strains are: SMR8843, *rpoE*::Tn[pYYF] (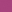); SMR8844 *rpoE*::Tn[pTrc] (▴); SMR8846, *rpoE*^+^[pYYF] (•); SMR8845, *rpoE*^+^[pTrc] (♦). B. Growth curve following IPTG addition. Symbols as in (A).

We find that *rpoE2072*::Tn*10*dCam cells do not induce the σ^E^ response to a stress peptide signal. We tested whether the transposon insertion in P2 affected stress induction of *rpoE* specifically by using an *rpoH*P3::*lacZ* fusion that is dependent on σ^E^ for activity (Mecsas *et al*., 1993). β-Galactosidase activity was assayed over time following initiation of the σ^E^ stress response by induction of plasmid-borne YYF, a peptide homologous to the unfolded outer membrane protein-stress signal that activates DegS protease to degrade RseA (anti-σ^E^), liberating σ^E^ to induce transcription of the stress response genes (Walsh *et al*., 2003). Our data show that stress induction of σ^E^-regulon genes is impaired in the *rpoE2072*::Tn*10*dCam mutant as follows. Following IPTG induction of the YYF stress-signal peptide from plasmid pBA166, β-galactosidase activity expressed from the *rpoH*P3*–lacZ* fusion increased approximately fivefold in *rpoE*^+^ cultures coincident with an increase in growth (Fig. 2A and B), whereas in the *rpoE2072*::Tn*10*dCam strain, β-galactosidase activity increased only slightly and the cultures stopped growing (Fig. 2A and B). The data show an inability of *rpoE2072*::Tn*10*dCam cells to induce the σ^E^ stress response in response to inducer peptide.

We can understand the cessation of growth in the *rpoE2072* mutant upon induction of YYF (Fig. 2B) as follows. YYF is expected to activate all σ^E^-regulated promoters, including the promoter immediately upstream of the *rseA* anti-sigma factor gene, but not the transposon-disrupted *rpoE*P2. This would be expected to result in induction of *rseA* but not *rpoE*, shifting the σ^E^:RseA ratio to favour RseA and decreasing available σ^E^. We suggest that an inability to elevate transcription of the σ^E^-dependent P2 promoter of *rpoE* in response to stress may result in an imbalance between sigma factor and anti-sigma factor, leading to depletion of active σ^E^ and thus the observed cessation of growth, because σ^E^ function is required for growth (Hiratsu *et al*., 1995; Rouviere *et al*., 1995; De Las Penas *et al*., 1997b).

### Stress-induced mutagenesis defect of *rpoE2072*::Tn*10*dCam and null strains

**Fig. 3:**
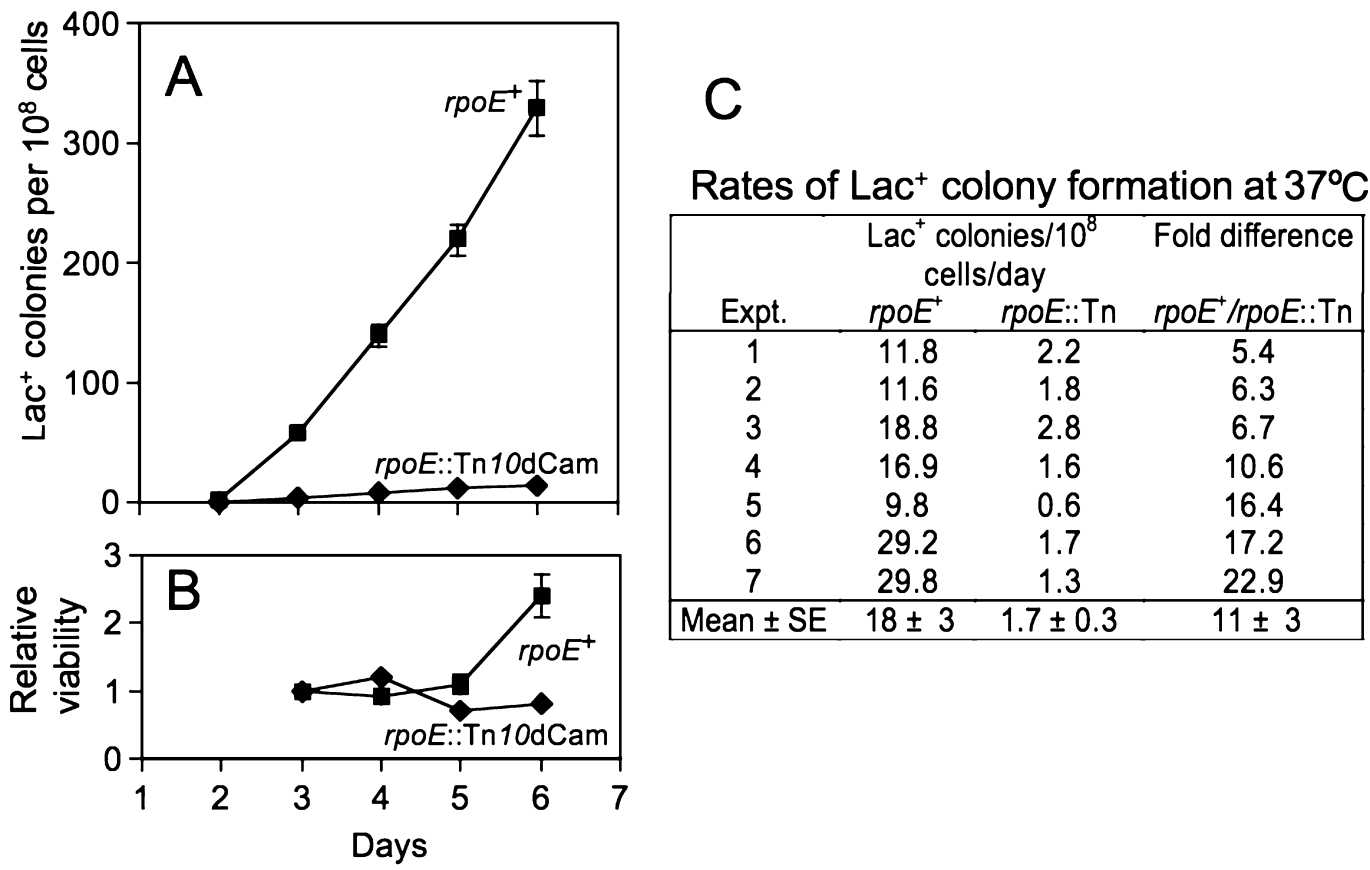
The *rpoE2072*::Tn*10*dCam mutation decreases stress-induced Lac^+^ reversion. Strains are *rpoE*^+^, SMR4562 (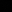); *rpoE2072*::Tn*10*dCam, SMR5236 (♦). A. Representative experiment performed at 30°C. Values are means ± one standard error of the mean (SEM) for eight independent cultures of each strain. Where not visible, error bars are smaller than the symbol. A second experiment at 30°C gave similar results. B. Relative viability of the Lac^-^ population monitored per Harris *et al*. (1996) beginning on the day after plating (day 1) for the experiment presented in (A). Values are means ± SEM for data from six selection plates. Because Lac^+^ mutant cells form colonies that are visible 2 days later (McKenzie *et al*., 1998), the day 3 Lac^+^ colony counts pertain to the day 1 viable cell measurements, and day 5 Lac^+^ colonies to the day 3 viable cells, etc. To make this comparison easier, we have shifted the viability data (B) 2 days rightward (the day 1 viability data are presented on day 3, etc.) for easier comparison with (A). C. Lac^+^ colony formation rates at 37° from multiple experiments. Lac^+^ colonies per day were calculated from colonies appearing from days 3–5 for seven independent stress-induced mutation assays and fold-difference between rates for SMR4562, *rpoE*^+^ and SMR5236, *rpoE2072*::Tn*10*dCam presented. Viability of all cultures was monitored per Harris *et al*. (1996). Mean ± SEM for the seven experiments is shown in last row of table. As observed previously, overall mutation rates are higher at 30°C than 37°C, although mutations that decrease mutagenesis do so similarly at both temperatures [Ponder *et al*. (2005) and A versus C].

First, quantitative stress-induced mutagenesis assays confirmed that *rpoE2072*::Tn*10*dCam decreases stress-induced Lac^+^ colony formation dramatically at 30°C (Fig. 3A) and 37°C (Fig. 3C). This decrease cannot be explained by net death of the population in that the overall survival of the population is decreased little during the stress-induced mutagenesis experiments (results range from no decrease, Fig. 3B, to at most a fourfold decrease at later time points in some experiments on amplification below). The Lac^+^ reversion rate from multiple experiments was approximately 11-fold lower in the *rpoE2072*::Tn*10*dCam than wild-type cells (Fig. 3C).

Second, an *rpoE* null mutation (in cells with extragenic suppressor mutations) causes a similar severe stress-induced mutagenesis defect (Fig. S2). Stress-induced mutagenesis assays were performed at 30°C, because even with its suppressor mutation, the *rpoE* null mutant is temperature-sensitive and cannot grow at 37°C (De Las Penas *et al*., 1997b; Alba and Gross, 2004). Nearly identically to the *rpoE2072*::Tn*10*dCam mutant, accumulation of stress-induced Lac^+^ mutant colonies was greatly reduced in the *rpoE* null mutant (Fig. S2). Similar results were obtained in another strain set in which the *rpoE*^+^, the *rpoE* null and the *rpoE2072*::Tn*10*dCam strains all carried Δ*ydcQ*, a known *rpoE* suppressor mutation (Button *et al*., 2007), to ensure their isogenicity (Fig. S2). Δ*ydcQ* increased the rate of accumulation of Lac^+^ mutant colonies in all strains but did not affect the relative reduction in the null mutant compared with the *rpoE*^+^ (Fig. S2). These results generalize the conclusion that functional σ^E^ is required for appearance of stress-induced mutants in the Lac assay.

Third, the apparent decrease in Lac^+^ colonies in the stress-induced mutagenesis assay could result either from a failure to form Lac^+^ mutations or from an inability of the Lac^+^ mutant colonies carrying an *rpoE* mutation to form colonies under the assay conditions. We performed reconstruction experiments the results of which show that cells carrying *rpoE2072*::Tn*10*dCam and a Lac^+^ mutation form colonies nearly normally under selective conditions (Table S1). Therefore, we conclude that formation of the mutants, not their subsequent growth on the selective medium, is impaired by the *rpoE2072*:*:*Tn*10*dCam allele.

The results shown in Tables S1 and S2 also address a specific hypothesis for why σ^E^ might be required for stress-induced mutagenesis. Lac^+^ stress-induced mutagenesis is associated with a cell subpopulation with transiently increased mutation rates (a hypermutable cell subpopulation) as seen by data showing that Lac^+^ mutants possess higher levels of other mutations throughout their genomes than stressed cells that did not become Lac^+^ (Torkelson *et al*., 1997; Rosche and Foster, 1999; Godoy and Fox, 2000), and that the mutagenesis process is not easily uncoupled from generation of these unselected ‘secondary’ mutations (Gonzalez *et al*., 2008). The σ^E^ stress response might have been required to allow normal growth rates of cells carrying additional unselected chromosomal mutations, perhaps by upregulating chaperones and proteases in response to resulting misfolded proteins. However, the data in Table S2 show that placing the *rpoE2072*::Tn*10*dCam allele into five different Lac^+^ revertants with accumulated unselected secondary mutations did not inhibit their colony-forming ability. The slightly longer times to form colonies overall reflect the lower growth temperature of 32°C used in these experiments (Table S2), whereas the slightly increased time for colony formation under lactose-selective conditions at 37°C (Table S1) appears to be the result of the *rpoE* mutation alone. Therefore, failure to ameliorate mutant proteins appears not to underlie the strong mutagenesis defect of the *rpoE2072*::Tn*10*dCam mutant (Fig. 3); rather, mutagenesis itself is implicated.

### Maintenance of gene amplification requires the σ^E^ response

**Fig. 4:**
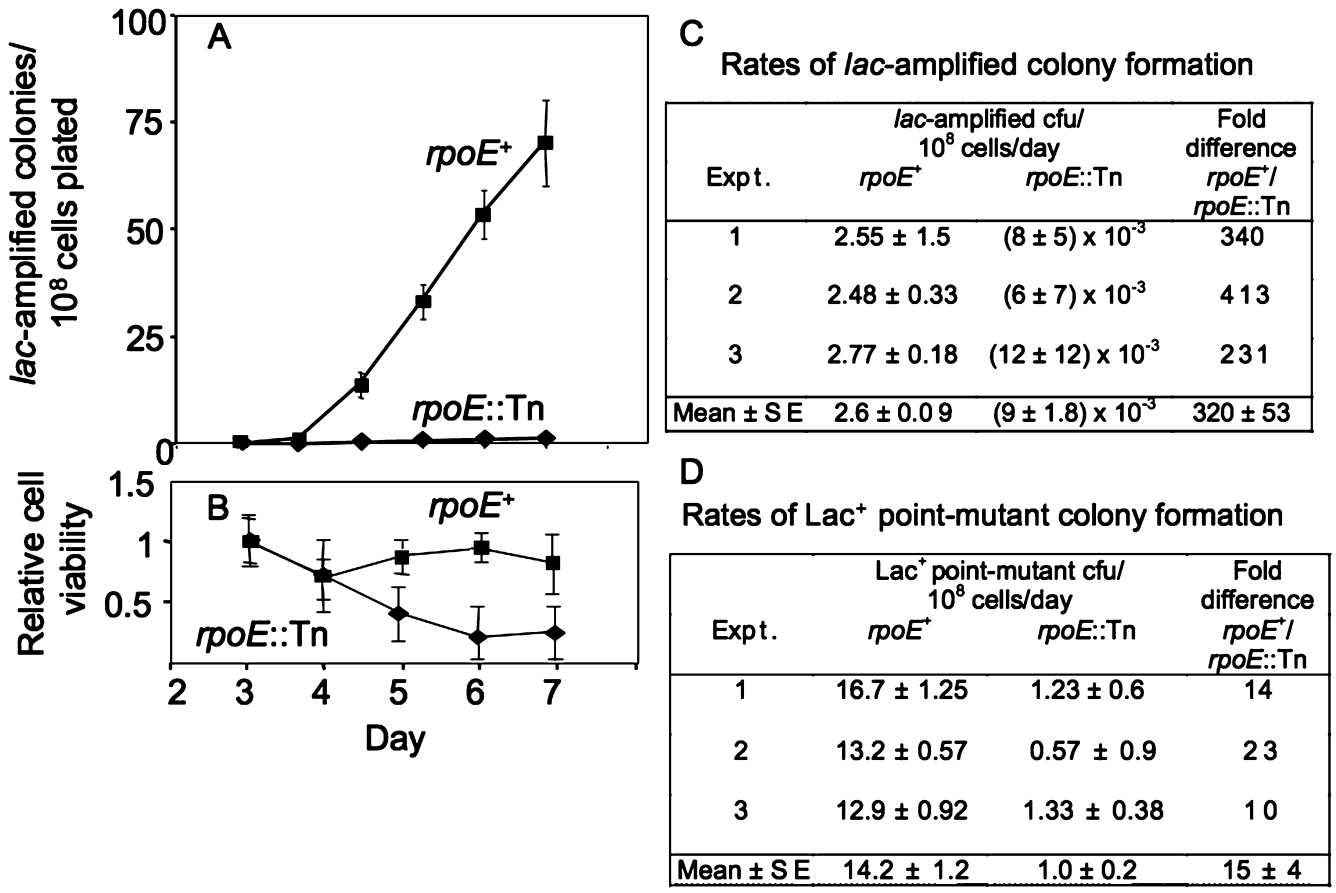
RpoE is required for stress-induced *lac* amplification. A.*lac*-amplified colonies, identified by sectored-colony morphology on LBH medium with X-gal, during a stress-induced mutagenesis experiment. Strains, *rpoE*^+^ SMR4562; *rpoE*::Tn SMR5236. Means for four cultures ± 1 SEM. Similar results were obtained from two additional experiments. B.Viable cell measurements were performed as described in Fig. 3. Means for four cultures ± 1 SEM. C.*lac* amplification rates from days 2–7 in three experiments. D.Lac^+^ point mutation rates from days 2–7 in three experiments. Point mutants were distinguished from *lac-*amplified clones by their pure blue colony morphology on LBH X-gal.

Stress-induced Lac^+^ colonies arise via either ‘point mutation’ (compensatory *lac* frameshift mutation) (Foster and Trimarchi, 1994; Rosenberg *et al*., 1994) or via amplification of the leaky *lac* allele, with *lac* amplification representing a minority of the Lac^+^ colonies until after day 8 of starvation on lactose plates (Hastings *et al*., 2000; Powell and Wartell, 2001). We found that accumulation of *lac-*amplified colonies was decreased 320-fold in the *rpoE2072*::Tn*10*dCam mutant relative to the *rpoE*^+^ strain (Fig. 4A and C). In these experiments, in which *lac-*amplified cfu were separated from the point mutants, the point mutation rate was decreased ∼15-fold by *rpoE2072*::Tn*10*dCam relative to *rpoE*^+^ (Fig. 4D). Although there is some loss of cell viability in the *rpoE* strain on later days in some experiments (e.g. Fig. 4B), the at most 75% (≤ 4-fold) loss of viability does not explain the ∼300-fold deficiency in accumulation of *lac-*amplified colonies.

Reconstruction of *rpoE2072*::Tn*10*dCam *lac-*amplified strains showed that the decrease in viable cfu (Fig. 4B) was not caused by slow growth, but rather an inability of *rpoE2072*::Tn*10*dCam strains to maintain amplification: first, on average, the transconjugants of *lac-*amplified DNA into *rpoE2072*::Tn*10*dCam recipients were 32-fold less able to form colonies on lactose medium than the *rpoE*^+^ transconjugants (Table 1, columns 2–4). Second, this lower recovery was not due to slower growth (5.1 ± 0.2 days for *rpoE*^+^ versus 4.9 ± 0.3 days for *rpoE2072*::Tn*10*dCam cells to form colonies, Table 1), or to lower efficiency of conjugation (Table 1, last two columns). Third, when F′ factors carrying a Lac^+^ point mutation were conjugated into *rpoE*^+^ and *rpoE2072*::Tn*10*dCam F^-^ cells (Table 1), there was no difference in conjugation efficiency between *rpoE*^+^ and *rpoE2072*::Tn*10*dCam strains, showing that the poor plating efficiency of *rpoE* cells plated on lactose is unique to amplification (Table 1). Thus, we conclude that this decrease represents a failure to maintain the amplified arrays. In addition to the ∼30-fold defect in maintenance of amplified arrays (Table 1) there is a variable ≤ 75% (≤ 4-fold) decrease in viable cell counts of the *rpoE2072*::Tn*10*dCam in late days in some experiments (Fig. 4B). These two factors can account for 30 × 4 = about 120-fold of the decrease observed in accumulation of *lac-*amplified cfu during stress-induced mutagenesis experiments. Because the decrease in accumulation of stress-induced *lac-*amplified colonies is ∼300-fold (Fig. 4C), it appears likely that, in addition to inability to maintain gene amplifications once formed, there was also a decrease in amplification formation caused by the *rpoE2072*::Tn*10*dCam mutation.

**Table 1:** Decreased ability to maintain *lac* amplification in *rpoE2072*::Tn*10*dCam cells.

	Transfer of *lac*-amplified DNA (Lac^+^ transconjugants/total transconjugants, mean ± SEM)^a^			Transfer of Lac^+^ point mutations (Lac^+^ transconjugants/total transconjugants, mean ± SEM)^b^			Average days to Lac^+^ colony formation of *lac* amplification carriers (mean ± SEM)^c^		Efficiency of conjugation (Pro^+^ Tet^R^ transconjugants/recipient cell)^d^	
Expt. No.	*rpoE*^+^	*rpoE*::Tn	*rpoE*^+^/*rpoE*::Tn	*rpoE*^+^	*rpoE*::Tn	*rpoE*^+^/ *rpoE*::Tn	*rpoE*^+^	*rpoE*::Tn	*rpoE*^+^	*rpoE*::Tn
1	0.20 ± 0.02	(3.2 ± 0.8) × 10^−3^	63	1.05	0.65	1.62	4.6 ± 0.5	5.0 ± 0.3	1.9 × 10^−3^	2.9 × 10^−3^
2	0.67 ± 0.05	(8.8 ± 1.8) × 10^−2^	7.7	0.99	0.90	1.10	5.3 ± 0.2	4.9 ± 0.2	1.8 × 10^−3^	1.4 × 10^−3^
3	0.53 ± 0.07	(2.1 ± 0.7) × 10^−2^	25	0.93	0.83	1.12	5.3 ± 0.2	4.9 ± 0.1	1.4 × 10^−3^	0.96 × 10^−3^
Mean ± SEM	0.47 ± 0.14	3.7 × 10^−2^ ± 2.6 × 10^−2^	32 ± 16	0.99 ± 0.04	0.79 ± 0.07	1.3 ± 0.17	5.1 ± 0.2	4.9 ± 0.3	1.7 × 10^−3^ ± 0.2 × 10^−3^	1.8 × 10^−3^ ± 0.6 × 10^−3^

a F′ plasmids carrying *lac*-amplified DNA and a *pro*^+^ marker were conjugated from donor strains PJH18, PJH51, PJH64, PJH69 and PJH74 into Δ(*lac-pro*) Tet^R^ recipients carrying either *rpoE*^+^ (PJH479) or *rpoE2072*::Tn*10*dCam (PJH480) and the ability of the transconjugants to form colonies was determined on minimal lactose Tet medium (selecting the multiple *lac* copies, Pro^+^, and the recipient Tet^R^ marker) and on minimal Tet medium (selecting Pro^+^ Tet^R^ transconjugants whether or not they maintain the *lac*-amplified DNA). The ratios of amplification-bearing (Lac^+^ Pro^+^ Tet^R^) to total (Pro^+^ Tet^R^) are shown. Transfer of plasmids carrying *lac* amplification into *rpoE2072*::Tn*10*dCam cells is 32-fold lower than into *rpoE*^+^ cells.

b Control experiments show no such bias against conjugation of Lac^+^ point mutations.

c Control experiments show no meaningful difference in the number of days required to form colonies of *lac* amplification bearers between *rpoE*^+^ and *rpoE2072*::Tn*10*dCam cells in reconstruction experiments discussed in the text.

d Control experiments show similar efficiencies of conjugation for these strains when amplification is not selected.

### σ^E^ induction is not generally mutagenic in growing cells

Fluctuation tests were performed to determine whether induction of the σ^E^ response provokes mutagenesis generally in growing cells, as, for example, induction of the RpoS regulon does (Yang *et al*., 2004). We measured mutation to rifampicin resistance, which occurs by any of a few specific base-substitution mutations in the *rpoB* gene (Jin and Gross, 1988), and used the plasmid-borne gene for the YYF peptide to induce expression of σ^E^-regulated promoters in *rpoE*^+^ cells. Expression of YYF had no effect on mutation rate per generation indicating that the σ^E^ response does not provoke mutagenesis in general but its requirement is specific for stress-induced mutagenesis (Table S3).

### Recombination proficiency in *rpoE2072*::Tn*10*dCam cells

σ^E^ upregulates transcription of homologous-recombination genes *recB*, *recD*, *recJ* and *recR* (Rezuchova *et al*., 2003; Kabir *et al*., 2005; Rhodius *et al*., 2006). Because *recB* mutants (Harris *et al*., 1994) and *recD recJ* double mutants (Foster and Rosche, 1999) are defective in stress-induced mutagenesis, possible loss of homologous-recombination proficiency in *rpoE2072*::Tn*10*dCam cells might explain their reduced stress-induced mutagenesis. However, first, whereas *recB* single mutants and *recD recJ* double mutants are hypersensitive to ultraviolet (UV) irradiation (Lovett *et al*., 1988), *rpoE2072*::Tn*10*dCam cells are not (Fig. S3), implying that levels of RecB, RecD and RecJ are not, in general, drastically reduced in this strain. These experiments cannot rule out insufficiency of RecB, RecD or RecJ under a particular stress condition that elicits a σ^E^ response. Second, quantitative Hfr-mediated conjugation experiments (Table S4) and quantitative P1 transduction experiments (Fig. S4) showed the *rpoE2072:*:Tn*10*dCam mutant to be as recombination-proficient as its isogenic *rpoE*^+^ parent. These results cannot exclude a possible recombination defect specific to recombination during cellular stress such as may occur during stress-induced mutagenesis. Third, if stress-induced mutagenesis required wild-type stress-inducible σ^E^ solely to produce sufficient levels of RecB, then we would expect stress-induced mutagenesis to be σ^E^-independent if a different homologous-recombination (HR) pathway was used to substitute for RecBC. Poteete *et al*. showed that the phage λ Red HR system can substitute for *recBC* in stress-induced mutagenesis in the Lac assay (Poteete *et al*., 2002). As shown previously (Poteete *et al*., 2002), mutation rate was stimulated more than eightfold in Δ*recBCD*::*red*^+^ relative to isogenic *recBC*^+^ cells (Fig. S5A). However, functional RpoE was still strongly required for stress-induced mutagenesis in Δ*recBCD*::*red*^+^ cells (Fig. S5A). Therefore, the primary cause of the mutagenesis defect of *rpoE* cells is not deficient expression of *recB* or *recD*. Fourth, *E. coli recJ* mutant cells are not impaired in stress-induced mutagenesis (Harris *et al*., 1994; Foster and Rosche, 1999), and we find that stress-induced mutagenesis was unchanged in the *recJ* or *recR* single mutants as well as in the *recJ recR* double mutant relative to the isogenic *rec*^+^ strain (Fig. S5B). Therefore, diminished RecJ and/or RecR production is not the cause of the stress-induced mutagenesis defect of *rpoE* cells.

### The role of the σ^E^ response in mutagenesis is independent of SOS and DinB upregulation, cytoplasmic heat-shock- and RpoS-stress responses

We wished to distinguish whether the σ^E^ response is an independent stress input into mutation, or feeds into the SOS, RpoS or cytoplasmic heat-shock stress responses. We first examined its effect on error-prone DNA polymerase, Pol IV or DinB, which is required for DSB-associated stress-induced point mutagenesis (Foster, 2000; McKenzie *et al*., 2001) and is upregulated transcriptionally by both the SOS (Kim *et al*., 1997; Courcelle *et al*., 2001) and RpoS (Layton and Foster, 2003) responses. *dinB* upregulation is the only role of the SOS response in stress-induced mutation (Galhardo *et al*., 2009). Although *dinB* upregulation by σ^E^ has not been reported (Rhodius *et al*., 2006), a modest increase might have been overlooked.

**Fig. 5:**
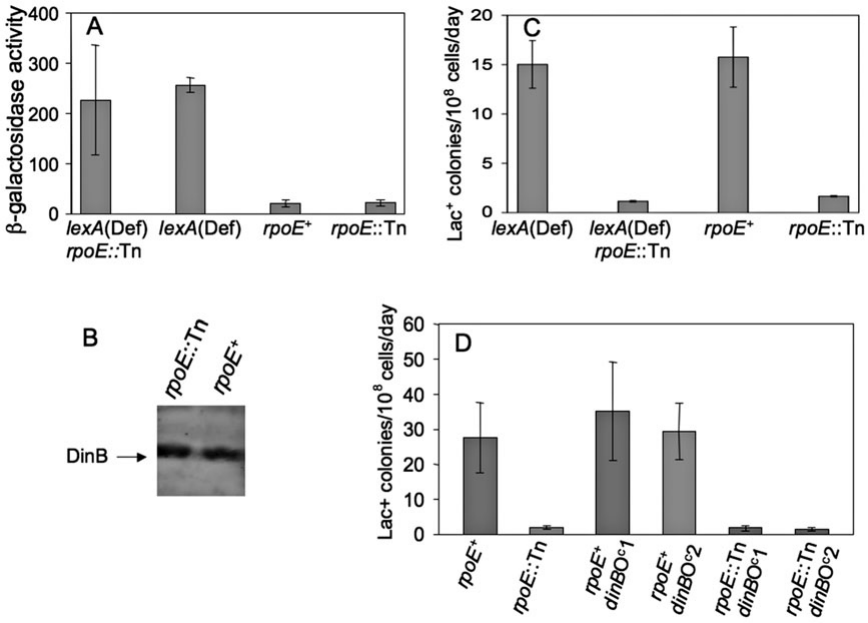
The role of the σ^E^ response in stress-induced mutagenesis is independent of SOS induction and *dinB* upregulation. A. Expression from the *dinB* promoter is not reduced in *rpoE* mutant cells. β-Galactosidase activity assayed in *rpoE*^+^ and *rpoE2072*::Tn*10*dCam cells carrying a plasmid-encoded P*_dinB_*::*lacZ* fusion. Strains are: *lexA*(Def)*rpoE*::Tn, SMR10475; *lexA*(Def), SMR10474; *rpoE*^+^, SMR10472; *rpoE*::Tn, SMR10479. β-Galactosidase activity is expressed as Miller units per 0.5 ml culture and is the average of two experiments ± range. B. DinB protein levels are not reduced in *rpoE2072*::Tn*10*dCam cells. Western immunoblot of *rpoE2072*::Tn*10*dCam (*rpoE*::Tn, SMR5236) and *rpoE*^+^ (SMR4562) cells using antibodies against DinB. Proteins separated by SDS-PAGE were blotted to PVDF membranes and reacted with antibodies as described in *Experimental procedures*. A separate experiment gave similar results. C. Constitutive expression of SOS/LexA regulon genes does not alleviate the requirement for an inducible σ^E^ response in stress-induced mutagenesis. Rates of stress-induced Lac^+^ colony formation at 37°C calculated from colonies arising from days 3–5 from three separate experiments. Mean ± SEM. Strains are: *lexA*(Def), SMR10369; *lexA*(Def)*rpoE*::Tn, SMR10370; *rpoE*^+^, SMR4562; *rpoE*::Tn, SMR5236. *lexA*(Def) strains also carry mutations in *sulA* (required for cell viability) and *psiB* (inactivating an SOS-upregulated inhibitor of mutation) per McKenzie *et al*. (2000). D. SOS-induced levels of DinB do not substitute for a stress-inducible σ^E^ in stress-induced mutagenesis. Experimental details as in (C). Strains are: *rpoE*^+^, SMR4562; *rpoE*::Tn, SMR5236; *dinB*O^c1^, SMR10464; *dinB*O^c2^, SMR10465; *rpoE*::Tn *dinB*O^c1^, SMR10466; *rpoE*::Tn *dinB*O^c2^, SMR10467.

First, we found that σ^E^ does not generally upregulate *dinB* transcription (Fig. 5A). We measured β-galactosidase activity in cells carrying a plasmid-borne *dinB* promoter fused to *lacZ* in *rpoE*^+^ and *rpoE2072*::Tn*10*dCam cells. No difference in activity was observed in either SOS-proficient *lexA*^+^ or *lexA*(Def) strains in which the SOS response is constitutively derepressed, suggesting that *dinB* transcription is not controlled by σ^E^ (Fig. 5A). Second, Western immunoblot analyses showed that DinB protein levels were unchanged in the *rpoE2072*::Tn*10*dCam mutant relative to *rpoE*^+^ cell extracts (Fig. 5B). Third, we asked whether the σ^E^ stress response might function by simply activating the SOS response, which is required for stress-induced mutagenesis (McKenzie *et al*., 2000), as follows. *lexA*(Def) (null) mutants are constitutively derepressed for the SOS/LexA regulon genes resulting in higher levels of SOS-controlled proteins including DinB (Friedberg *et al*., 2005). The factor(s) regulated by σ^E^ appear not to be DinB or any other protein induced by the SOS response because the *lexA*(Def) allele did not restore the ability to mutate to *rpoE2072*::Tn*10*dCam cells (Fig. 5C). Fourth, we used two operator-constitutive mutations of *dinB* that produce SOS-induced levels of DinB constitutively (Galhardo *et al*., 2009), and found that these do not restore normal levels of mutagenesis to *rpoE2072*::Tn*10*dCam cells (Fig. 5D), although they do to SOS-induction-defective cells (Galhardo *et al*., 2009). These results show that SOS induction and *dinB* expression are not the limiting factor for stress-induced mutagenesis in the *rpoE2072*::Tn*10*dCam cells defective for inducing the σ^E^ response.

**Fig. 6:**
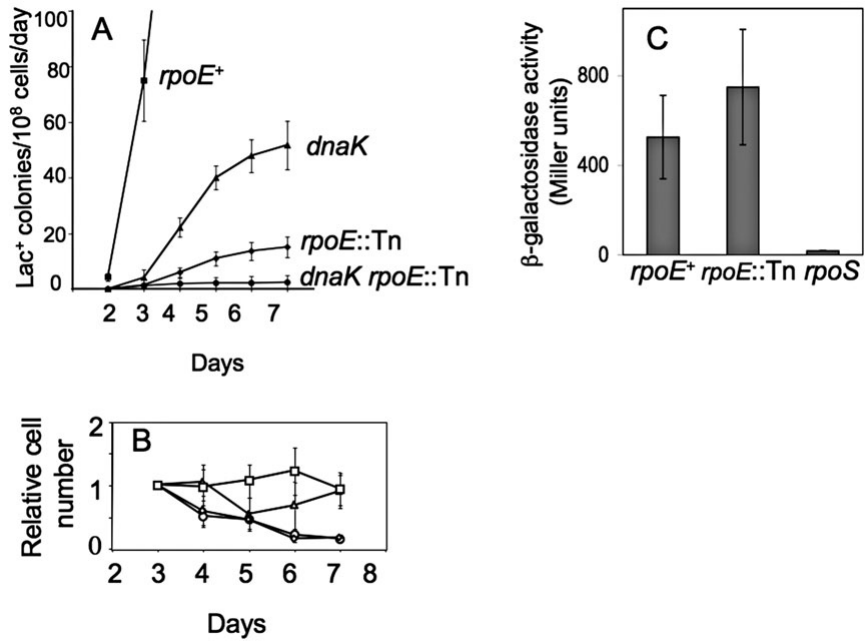
*rpoE2072*::Tn*10*dCam inhibits stress-induced mutagenesis independently of effects on the σ^32^ cytoplasmic heat-shock- or RpoS-stress responses. A. Constitutive activation of the σ^32^ response genes in *dnaK* mutant cells does not substitute for a functional σ^E^ response in stress-induced mutagenesis. Strains are: SMR4562 *rpoE*^+^ (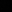); SMR8862 *dnaK* (▴); SMR5236 *rpoE*::Tn *(*♦); SMR8863 *rpoE*::Tn *dnaK* (•). Assay was performed at 37°C as described in *Experimental procedures*. Values are means ± one SEM for six independent cultures of each strain in one experiment. Three experiments gave similar results. B. Viability of all cultures was monitored per Harris *et al*. (1996). Strains and symbols are as in (A) but with open symbols. C. Activity of the RpoS-dependent *katE* promoter is not diminished by *rpoE2072*::Tn*10*dCam. β-Galactosidase activity from a *katE*::*lacZ* fusion was measured in saturated LBH cultures in strains SL590, *rpoE*^+^; SMR8919, *rpoE2072*::Tn; and CH1761, *rpoS*::FRTKan. The means ± range of two experiments are shown. Error bars are too small to see for the *rpoS* strain.

The σ^E^ stress response upregulates σ^32^, the transcriptional activator of the cytoplasmic unfolded-protein or heat-shock response, which in turn increases transcription of the genes encoding the GroELS chaperone (Lund, 2001), which is required for efficient stress-induced Lac reversion (Layton and Foster, 2005). We tested whether the requirement for the σ^E^ response induction in mutagenesis was in fact a requirement for inducing the σ^32^ regulon by examining *dnaK756* mutant cells, in which the σ^32^ regulon is expressed at induced levels constitutively (Straus *et al*., 1990). The data show that the *dnaK* mutation does not substitute for a functional σ^E^ response as follows (Fig. 6). First, the *dnaK* mutation decreases stress-induced Lac reversion (Fig. 6A). However, a functional RpoE response is still required for the mutagenesis that remains, as seen by the severe decrease in mutagenesis in the *dnaK rpoE2072:*:Tn*10*dCam double mutant relative to the *dnaK* mutant cells (Fig. 6A). These data indicate that the σ^E^ stress response provides function(s) for mutagenesis other than or in addition to those upregulated by the σ^32^-activated cytoplasmic heat-shock response.

The general- or stationary-phase-stress response controlled by σ^S^ (RpoS) is required for both stress-induced point mutagenesis (Layton and Foster, 2003; Lombardo *et al*., 2004) and amplification (Lombardo *et al*., 2004); however, two lines of evidence imply that a defect in inducing the RpoS stress response is not the primary cause of diminished mutagenesis in *rpoE* mutant cells. First, because *katE* is induced RpoS-dependently in stationary phase (Mulvey *et al*., 1990), we assayed β-galactosidase activity in *rpoE*^+^ and *rpoE2072:*:Tn*10*dCam saturated cultures that contained the *katE*::*lacZ* fusion. We find that *rpoE2072:*:Tn*10*dCam cells induce the fusion gene similarly to isogenic *rpoE*^+^ cells and more than the *rpoS* deletion mutant in which β-galactosidase activity is severely reduced (Fig. 6C). Second, the requirement for *rpoS* in stress-induced mutagenesis is not mitigated by provision of DNA DSBs using a restriction enzyme *in vivo*, indicating that RpoS acts downstream of the production of DSBs in the mutagenic mechanism (Ponder *et al*., 2005). We show in the following section that much of the requirement for σ^E^ is alleviated by introduction of enzyme-generated DSBs.

### Regulation of DSB formation by σ^E^

A fundamental requirement for stress-induced mutagenesis is a DSB, which, in the F′, is thought to originate most frequently from a single-strand nick made at *oriT* by the F-encoded TraI endonuclease (Foster and Trimarchi, 1995; Rosenberg *et al*., 1995). This single-strand break is thought to become a DSB during replication (Kuzminov, 1995; Rosenberg *et al*., 1995; Rodriguez *et al*., 2002). TraI is required for stress-induced mutation of *lac* in the F′ but this requirement can be bypassed entirely, and mutagenesis stimulated an additional ∼70-fold more, by DSBs made near *lac* by the I-SceI double-strand endonuclease *in vivo* (Ponder *et al*., 2005). I-SceI-induced DSBs made near *lac* increased mutation rate ∼6000-fold in the absence of *traI* and ∼70-fold in its presence (Ponder *et al*., 2005). Unlike TraI, the requirements for DSB-repair proteins, an inducible SOS response, RpoS and DinB could not be substituted for by I-SceI cuts. The substitution of I-SceI cuts specifically for TraI supports the model that the role of TraI in mutagenesis is the ultimate generation of a DSB (Ponder *et al*., 2005).

**Fig. 7:**
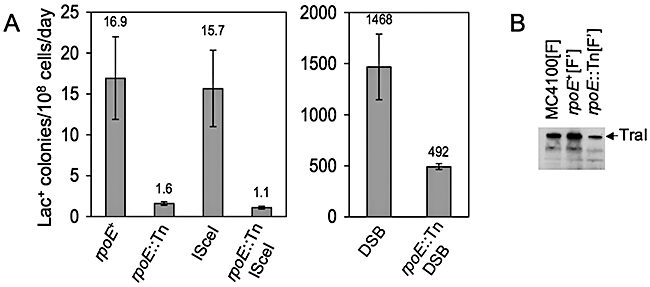
I-SceI-generated DSBs relieve much of the *rpoE2072*::Tn*10*dCam defect in stress-induced mutagenesis. A. Stress-induced mutation rates in the presence of I-SceI generated DSBs. Expression of I-SceI enzyme in cells with no I-SceI cutsite (left panel) does not affect the requirement for functional *rpoE* in stress-induced mutagenesis. However when I-SceI is expressed in cells with a cutsite near *lac* (DSBs, right panel), the *rpoE2072*::Tn*10*dCam phenotype is greatly reduced. Rates are Lac^+^ cfu per 10^8^ cells per day between days 3 and 5 and represent the averages of four experiments ± SEM. Experiments were performed at 37°C as described in *Experimental procedures*, and viability monitored per Harris *et al*. (1996). Strains are: *rpoE*^+^, SMR4562; *rpoE*::Tn, SMR5236; *rpoE*^+^ I-SceI (enzyme, no cutsite), SMR6276; *rpoE*::Tn I-SceI (enzyme, no cutsite), SMR9191; *rpoE*^+^ DSB (enzyme + cutsite), SMR6280; and *rpoE*::Tn DSB (enzyme + cutsite), SMR10168. B. Western immunoblot using antibodies against TraI. Proteins separated by SDS-PAGE as described in *Experimental procedures* were blotted to nitrocellulose membranes and probed with anti-TraI. MC4100 [F^+^]; *rpoE*^+^[F′], SMR4562; *rpoE*::Tn[F′], SMR5236. Two separate experiments gave similar results.

We found that I-SceI-induced DSBs near *lac* could relieve much of the mutagenesis defect caused by *rpoE2072:*:Tn*10*dCam (Fig. 7A). First, in the left panel in Fig. 7A, we see that *rpoE2072:*:Tn*10*dCam depresses the mutation rate 15-fold in cells that do not make I-SceI-induced DSBs because they have either only the chromosomal inducible I-*Sce*I gene but no cutsite (ISceI and *rpoE*::Tn ISceI), or have neither enzyme nor cutsite. Second, when DSBs were induced in the *rpoE2072:*:Tn*10*dCam mutant (Fig. 7A, right panel), the stress-induced Lac^+^ reversion rate increased nearly 500-fold relative to the control strain that expressed I-SceI in the absence of the I-SceI cutsite (Fig. 7A, left panel). In the DSB-inducing strain (Fig. 7A, right panel), the *rpoE2072:*:Tn*10*dCam mutation caused only a threefold depression of mutation rate, compared with its 15-fold defect in the absence of I-SceI-made DSBs (Fig. 7A, left panel). This constitutes an ∼80% alleviation of the *rpoE2072:*:Tn*10*dCam mutagenesis defect (3/15 = 20% of the mutagenesis defect remaining). Thus much of the requirement for the σ^E^ response in stress-induced mutagenesis is a requirement for creation of the DSBs that provoke mutagenesis, implying that the σ^E^ response promotes DSB formation in F. This numerical comparison assumes that the mutation mechanism is the same with I-SceI cuts as with TraI. In all ways testable, this was shown to be so previously: *lac* mutation sequence spectrum, requirements for RecA, RecB, Ruv, SOS, RpoS and DinB, and fraction amplified and point mutant (Ponder *et al*., 2005). Thus, the comparison appears justified. Western analyses show that TraI protein levels are reduced only about 30 ± 0.6% in the *rpoE2072*::Tn*10*dCam mutant relative to isogenic *rpoE*^+^ cells (Fig. 7B). Thus, although DSB formation appears to be limiting in the *rpoE2072*::Tn*10*dCam background, it is probably not due to lowered expression of the F plasmid *traI*. We cannot rule out an effect on TraI activity; however, unaltered conjugation frequency in the *rpoE2072*::Tn*10*dCam mutant (data not shown) argues against this possibility. Other possibilities are considered below.

## Discussion

### Separation of the essential and stress–response functions of σ^E^

One interesting aspect of the data presented is the discovery that *rpoE2072*::Tn*10*dCam, a mutation that allows constitutive expression but not induction of the σ^E^ stress response by the YYF inducing peptide (Fig. 2), retains viability in the absence of unlinked suppressor mutations (Fig. S1). This implies that the essential function of σ^E^ relates to one or more of the functions it controls constitutively, not to transient expression of the stress response. The *rpoE2072*::Tn*10*dCam allele is likely to provide a useful reagent for future genetic studies of the effects of loss of the stress response, which can now be achieved both specifically, without simultaneous loss of the essential function, and cleanly, in cells that do not also carry suppressor mutations.

### Control of genomic instability by the σ^E^ stress response and σ^E^-activating stressors

The data presented show that the stress–response function of σ^E^ is required for stress-induced point mutagenesis (Figs 3 and 4D, Tables S1 and S2) and gene amplification (Fig. 4, Table 1). Moreover σ^E^ constitutes an independent stress and stress–response input to mutation, independent of known SOS and σ^S^ and postulated σ^32^ heat-shock response involvement (Figs 5–7). Therefore, first, stress-induced point mutagenesis and amplification are controlled by multiple independent stress responses: the SOS DNA-damage (McKenzie *et al*., 2000), RpoS general-stress (Layton and Foster, 2003; Lombardo *et al*., 2004) and σ^E^ responses for point mutation, and RpoS (Lombardo *et al*., 2004) and σ^E^ for amplification. Input of multiple stress responses is important in regulating the circumstances under which cells increase genetic diversity and their potential to evolve. Second, the results indicate that the σ^E^ response influences genome (in)stability generally.

Although both σ^E^ and σ^S^ responses are necessary for the genomic instability studied here, many stressors are expected to activate both, including starvation (via ppGpp) (Costanzo and Ades, 2006; Costanzo *et al*., 2008), but also many antibiotics, which may trigger membrane stress (such as β-lactams) and all of which appear to induce reactive oxygen species (reviewed, Kohanski *et al*., 2010) and so oxidative stress, an inducer of σ^S^. The coupling of mutagenesis to antibiotic stressors is a potentially serious problem with resistance mechanisms instigated by the antibiotics (Cirz and Romesberg, 2007; Galhardo *et al*., 2007; Lopez and Blazquez, 2009; Cohen and Walker, 2010; Kohanski *et al*., 2010).

### Role of σ^E^ response in stress-induced mutagenesis

One major role of the σ^E^ response in stress-induced mutagenesis is apparent: ∼80% of its function is substituted by an I-Sce-induced DSB (Fig. 7), implying that somehow the σ^E^ response promotes TraI-generated double-strand-end (DSE) formation, for which I-SceI also substitutes (Ponder *et al*., 2005). Previous work indicates that the point mutagenesis occurs in acts of error-prone DSB repair, and that in the F′, most of the DSBs originate from the action of TraI endonuclease (Ponder *et al*., 2005). The breaks are repaired non-mutagenically in unstressed cells and mutagenically, using DinB error-prone DNA polymerase if RpoS is induced either by stress or artificially (Ponder *et al*., 2005). The σ^E^ response could promote either the nicking by TraI or the replication into that nick. Although TraI levels were affected only modestly in the *rpoE2072*::Tn*10*dCam mutant (Fig. 7B), it is possible that replication or specifically F replication might be affected by σ^E^, and the effect of σ^E^ might be F-specific. The mechanism of the remaining ∼20% of the σ^E^-response effect is unknown. Given that one of the ways that the σ^E^ response is induced is via starvation using ppGpp and the stringent response (Costanzo and Ades, 2006; Costanzo *et al*., 2008), it might, like RpoS, facilitate the switch to mutagenic repair of DSBs under stress.

### Alternative amplification-mutagenesis model

Others favour a model of mutation in the Lac system that does not involve stress or stress responses but instead invokes growth of cells carrying an amplified *lac* array, which produces more β-galactosidase activity from the weakly functional mutant *lac* gene (Roth *et al*., 2006; Roth, 2010). In this model, when about 10^5^ cells with many *lac* copies are present in a microcolony, a Lac^+^ point mutation occurs spontaneously, and the point mutant then overgrows the colony. We do not favour this model for several reasons. First, its prediction that point mutants arise from *lac-*amplified clones was contradicted by experiments showing that: (i) Lac^+^ microcolonies, as early as the two-cell stage, are pure point mutants, not mostly amplified with point mutants arising later – point mutants did not arise in amplified young colonies (Hastings *et al*., 2004), (ii) *lac*-amplified microcolonies do not produce point mutants efficiently under selective conditions (Hastings *et al*., 2004), and (iii) mutation of the DNA polymerase I gene obliterates amplification without diminishing point mutation (Hastings *et al*., 2004; Slack *et al*., 2006). This would be impossible if amplification were a precursor to point mutation, as the model specifies. As far as we are aware, alternative interpretations of these data have not been offered. Other data also contradict this model (Stumpf *et al*., 2007). Second, the fact that three stress responses are required for point mutation [SOS (McKenzie *et al*., 2000), RpoS/σ^S^ (Layton and Foster, 2003; Lombardo *et al*., 2004) and σ^E^] and two for amplification [σ^S^ (Lombardo *et al*., 2004) and σ^E^] is not compatible with the amplification model, which does not involve stress or stress responses. Neither are results, reviewed above, that showed that DSB repair switches to a mutagenic mode using DinB if RpoS is expressed, even in the absence of any external stress or selection (Ponder *et al*., 2005). We do not know of alternative interpretations for those data, and favour their obvious interpretation: that stress responses upregulate mutagenesis when cells are stressed.

### Mutation as a stress response and the regulation of evolvability

The coupling of mutagenesis to stress responses means that cells turn up mutation rate specifically when they are maladapted to their environment, i.e. are stressed, potentially accelerating evolution specifically then. This can be a powerful device for adaptation to adversity including antibiotics and host defences in bacteria (McKenzie and Rosenberg, 2001; Cirz *et al*., 2005; Galhardo *et al*., 2007), and hypoxia (Bindra *et al*., 2007; Huang *et al*., 2007) and chemotherapeutic drugs in developing cancers. More than 80% of *E. coli* natural isolates respond to stress with mutagenesis, and modelling supports the potential benefits in enhanced evolvability of this response (Bjedov *et al*., 2003).

The requirement for (at least) three stress responses for point mutagenesis (SOS, σ^S^ and σ^E^) and two for amplification (σ^S^ and σ^E^) implies that cells do not instigate potentially dangerous programmes of genomic instability until they sense multiple independent stressors. Similarly in *B. subtilis*, both the stringent response (Rudner *et al*., 1999b) and ComK-controlled competence stress response (Sung and Yasbin, 2002) are required for starvation-induced mutagenesis. In *Salmonella*, bile-induced resistance mutagenesis requires both SOS and σ^S^ responses (Prieto *et al*., 2006; J. Casadesus, pers. comm.). Coupling genome instability to more than one stress response, any of which might not be turned on in all cells in a population, may further restrict or regulate mutagenesis to a cell subpopulation (Galhardo *et al*., 2007; Gonzalez *et al*., 2008), providing one way to achieve a potential bet-hedging mechanism such as is seen in bistable subpopulations critical to many bacterial survival strategies (Veening *et al*., 2008). These are highly regulated programmes, exquisitely tuned to cellular stresses, which regulate mutagenesis, and thus the ability to evolve, temporally. Understanding and targeting the regulatory components is likely to provide powerful new antibiotic strategies.

## Experimental procedures

### Bacterial strains and growth conditions

Strains used are listed in Table S5. Standard genetic techniques were used in strain constructions (Miller, 1992). All M9 minimal media (Miller, 1992) had carbon sources added at 0.1% and thiamine (vitamin B1) at 10 µg ml^−1^. LBH medium is per, for example, Torkelson *et al*. (1997). Antibiotic and other additives were used at the following final concentrations: ampicillin, 100 µg ml^−1^; chloramphenicol, 25 µg ml^−1^; kanamycin, 50 µg ml^−1^; tetracycline, 10 µg ml^−1^; rifampicin, 100 µg ml^−1^; 5-bromo-4-chloro-3-indolyl-β-d-galactoside (X-gal), 40 µg ml^−1^; sodium citrate, 20 mM.

### RpoE reconstruction experiments

Lac^+^ strains carrying *rpoE2072*::Tn*10*dCam were constructed and their growth on lactose quantified (Table S1). The *rpoE* allele was moved by P1 transduction into three Lac^+^ day 5 stress-induced mutants that had been characterized with respect to colony-forming ability on lactose medium (Rosenberg *et al*., 1998). To measure the days required for colony formation on lactose minimal medium, growth and plating of cells was identical to that described for stress-induced mutagenesis experiments, including the addition of *lac*-deleted scavenger cells in the same numbers. Additionally, the *rpoE2072*::Tn*10*dCam allele was transferred by P1 transduction into five Lac^+^ stress-induced mutants known to carry secondary mutations (Torkelson *et al*., 1997) and the ability to form colonies in exact reconstruction of experimental selection conditions measured. These experiments were carried out at 32°C due to the temperature-sensitive phenotype of one of the mutants.

### Generation-dependent mutagenesis assays

Generation-dependent mutation rates to rifampicin resistance were measured in MG1655 cells harbouring either pBA166 or the vector pTrc99a. Single colonies grown on LBH-amp plates were inoculated into tubes containing 5 ml of LBH-amp broth with either 0.1% glucose or 0.1 mM IPTG to repress or induce expression of YYF from pBA166 respectively. Cultures were incubated overnight at 37°C prior to plating on LBH plates containing rifampicin. For each determination, 19 cultures were used and median mutant frequencies used to estimate mutation rates using a modified method of the median (Lea and Coulson, 1949; Von Borstel, 1978).

### Mutation assays, quantitative conjugation and P1 transduction

Stress-induced mutagenesis assays were performed as described at 30°C or 37°C unless otherwise indicated (Harris *et al*., 1996). SMR5236 cultures were concentrated 10-fold before plating to obtain sufficient numbers of Lac^+^ colonies. In some experiments amplification of the *lac* region was quantified as described (Hastings *et al*., 2004).

In reconstruction experiments for amplification, *rpoE*^+^ and *rpoE2072*::Tn*10*dCam F^-^ cells were plated under the conditions of stress-induced mutagenesis experiments after conjugation with the same set of five F′ factors carrying *lac* amplification. The ratio of donor to recipient cells was 20:1. Transconjugants were selected on M9 glycerol tetracycline medium, which selects for Tet^R^, carried in the chromosome of the recipient cells and for Pro^+^, bestowed by the F′ factor, and also on M9 lactose tetracycline medium, which selects for the recipient chromosome and the donor F′, as above, and also requires the maintenance of amplification of the *lac* locus. Thus the fraction of the colonies on glycerol medium that forms colonies on lactose gives a measure of the ability of cells of that genotype to support amplification.

Bacterial strains carrying the inducible DSB system consisting of a chromosomally encoded I-SceI enzyme and an F′ cutsite were maintained on medium containing 0.1% glucose to repress I-SceI synthesis (Ponder *et al*., 2005) and experiments performed as described (Ponder *et al*., 2005).

Quantitative conjugation and P1 transduction assays were performed as described (Lombardo *et al*., 2004). P1 stocks grown on SMR6263 were used in transduction experiments. Transductants were selected on LBH tetracycline citrate medium.

Co-transduction experiments (Fig. S1) were conducted with P1 grown on SMR11044 which has a kanamycin marker (*yfhH*::FRTKan) linked to *rpoE2072*::Tn*10*dCam. Following transduction, cells were plated on either LBH-kanamycin plates or LBH-chloramphenicol plates. Resistant colonies were then screened for the second antibiotic resistance. Co-transductant frequency was calculated as the number of screened antibiotic-resistant colonies per the number of selected antibiotic-resistant colonies. If the insertion in *rpoE* confers a null phenotype, the co-transductant frequency of chloramphenicol-resistant per kanamycin-resistant colonies would be expected to be close to zero.

UV sensitivity was determined in saturated overnight LBH cultures. Varying dilutions were spread on LBH plates, irradiated, and the plates immediately placed in the dark and incubated overnight at 37°C.

### β-Galactosidase assays and Western immunoblot analysis

For monitoring expression of a σ^E^-regulated gene, the *rpoE2072*::Tn*10*dCam allele was moved into strain CAG45114, containing an *rpoH*P3::*lacZ* fusion. For monitoring expression of an RpoS-controlled gene, *rpoE2072*::Tn*10*dCam was moved into SL590, containing a *katE*::*lacZ* fusion. The presence of the transposon insertion was verified by PCR amplification using primers flanking the insertion, RpoE-F (5′-CACTGGAAGGTGGACGACG) or RpoE-R (5′-GAGAAGTTACTGGCTGGTGG), in conjunction with an outward-reading primer (5′-GGTGGTGCGTAACGGCAAAAG) specific for Tn*10*dCam.

For β-galactosidase assays saturated LBH or M9-glucose cultures were diluted back 1:100 in fresh medium. For induction of peptide synthesis from pBA166, 1.0 mM IPTG was added to LBH cultures at OD_600_, of 0.1 and 0.5 ml aliquots were removed at various time intervals for β-galactosidase assays. Assays were performed as described (Miller, 1992). β-Galactosidase activity is expressed as a function of culture volume. For monitoring β-galactosidase activity of the *katE*::*lacZ* fusion throughout the growth phase, LBH cultures were inoculated from saturated overnight cultures. OD_600_ and β-galactosidase measurements were determined for each sample.

Western blots were performed with polyclonal antiserum against purified DinB as described (Galhardo *et al*., 2009). TraI was examined by immunoblot analysis using a 1:5 000 dilution of polyclonal antiserum against TraI as described (Will and Frost, 2006).
