# Supplementary material for: The σE stress response is required for stress-induced mutation and amplification in Escherichia coli
Source: Mol Microbiol. 2010 Jun 7;77(2):415–30. doi: 10.1111/j.1365-2958.2010.07213.x (PMC2909356; doi:10.1111/j.1365-2958.2010.07213.x)
Supplement: Supplementary file 1 [file mmi0077-0415-SD1.pdf]

Supporting information

Table S1. *rpoE2072*::Tn*10*dCam has little effect on speed of colony formation in reconstruction experiments.

Strain	Relevant Genotype	Average days to colony formation[a]	% viable cells forming Lac^+ colonies[a]
SMR3856	Lac^+	2.0 ± 0.1	103 ± 5
SMR3858	Lac^+	2.0 ± 0.1	95 ± 3
SMR3859	Lac^+	2.0 ± 0.1	103 ± 4
SMR8838	Lac^+ *rpoE*::Tn*10*dCam	2.5 ± 0.3	78 ± 8
SMR8839	Lac^+ *rpoE*::Tn*10*dCam	2.5 ± 0.2	82 ± 18
SMR8840	Lac^+ *rpoE*::Tn*10*dCam	2.4 ± 0.3	80 ± 15

[a] Values are the average of two to four separate growth experiments ± range. In each case, four independent cultures were used to determine days to form colonies on lactose minimal medium, in the presence of scavenger cells at 37°C, as described for the Lac^+ reversion assay (Experimental Procedures). In this and the following table, days = 24 hour intervals such that, *e.g.*, 2.5 days = 36 hours. % viable cells forming Lac^+ colonies refers to the fraction of cfu on M9 B1 lactose *versus* M9 B1 glycerol plates.

Table S2. Colony formation in reconstruction experiments of Lac^+ revertants with multiple mutations is unaffected by *rpoE2072*::Tn*10*dCam.

Strain[a] (*rpoE*$^+$ / *rpoE*::Tn)	Days to form colony	
	rpoE$^+$	***rpoE2072*::Tn*10*dCam**
SMR11033 / 11038	2.96 ± 0.10	3.11 ± 0.04
SMR11034 / 11039	3.0 ± 0.04	2.97 ± 0.08
SMR11035 / 11040	3.02 ± 0.02	3.02 ± 0.02
SMR11036 / 11041	3.00 ± 0.03	3.01 ± 0.01
SMR11037 / 11042	3.79 ± 0.14	3.71 ± 0.05

[a] Strains are Lac^+ revertants from stress-induced-mutation assays identified as carrying unselected secondary mutations (Torkelson *et al.*, 1997) and are isogenic with the exception of the *rpoE2072* allele. Data are the averages of two experiments ± range. Reconstruction experiments performed at 32°.

Table S3. σ^E-response induction does not affect rifampicin-resistance-mutation rate in growing cultures.

Strain	+IPTG		-IPTG	
	mutations/cell/ generation[a]	SEM	mutations/cell/ generation[a]	SEM
MG1655[pTrc99a]	1.3×10^{-9}	0.3×10^{-9}	8.7×10^{-10}	0.4×10^{-10}
MG1655[pYYF]	1.5×10^{-9}	0.9×10^{-9}	9.7×10^{-10}	0.6×10^{-10}

[a] Averages of three experiments. Mutation rates determined per Experimental Procedures.

Table S4. Conjugational recombination is unimpaired by *rpoE2072*::Tn*10*dCam.

**Recipient**	**Relevant genotype**	**Tet^R transconjugants / donor[a]**
FC36	*rpoE*$^+$	3.4 x 10^{-4} ± 3.8 x 10^{-5}
PJH427	*rpoE2072*::Tn*10*dCam	3.2 x 10^{-4} ± 1.3 x 10^{-4}

[a]Frequencies are tetracycline-resistant transconjugants obtained per donor cell from experiments with the tetracycline-resistance marker, *argE*86::*Tn*10, transferred from Hfr-donor strain BW6175 to recipient cells in mating mixes with donor cells limiting (≤10-fold fewer donors than recipients). Mean ± SEM of three experiments.

Table S5. *E. coli* strains and plasmids used in this study.

**Strain**	**Relevant genotype**	**Reference/Source**
BW6175	*argE*::Tn*10* Hfr point of origin :PO131	(Wanner, 1986)
CAG18495	*zih35*::Tn*10*	(Singer *et al*., 1989b)
CAG22216	*rpoE*::ΩCm (λ *rpoH*P3-*lacZ*)	(Rouviere *et al*., 1995)
CAG45114	MG1655 (λ *rpoH*P3-*lacZ*)	(Ades *et al*., 2003)
CH1761	594 *rpoS*::FRTKanFRT	C. Herman
FC29	Δ(*lac-proB*)XIII [F' *proAB*$^+$ Δ(*lacI-Z*)]	(Cairns & Foster, 1991)
FC36	Δ(*lac-proB*)XIII *thi ara* Rif^R F$^-$ λ$^-$	(Cairns & Foster, 1991)
FC40	Δ(*lac-proB*)XIII *thi ara* Rif^R [F'128 *proAB*$^+$ *lacIq lacI33*Ω*lacZ*]	(Cairns & Foster, 1991)
JW1433	*ydcQ*::FRTKanFRT	(Baba *et al*., 2006)
JW2545	*yfhH*::FRTKanFRT	(Baba *et al*., 2006)
MC4100[F$^+$]	carries wild-type F	Frost lab
MG1655	sequenced wild-type K12	*E. coli* Genetic Stock Center (Yale)
PJH18	SMR4562 carrying amplification of the *lac* region	(Hastings *et al*., 2000)
PJH51	SMR4562 carrying amplification of the *lac* region	(Hastings *et al*., 2000)
PJH64	SMR4562 carrying amplification of the *lac* region	(Hastings *et al*., 2000)
PJH69	SMR4562 carrying amplification of the *lac* region	(Hastings *et al*., 2000)
PJH74	SMR4562 carrying amplification of the *lac* region	(Hastings *et al*., 2000)
PJH427	FC36 *rpoE2072*::Tn*10*dCam	FC36 x P1 (SMR5236)
PJH479	SMR4562 F$^-$ *malB*::Tn*9* *zih35*::Tn*10*	spontaneous F$^-$ derivative of SMR533 x P1(CAG18495)
PJH480	SMR4562 F$^-$ *rpoE2072*::Tn*10*dCam *zih35*::Tn*10*	spontaneous F$^-$ derivative of SMR5236 x P1 (CAG18495)
SL471	JM105 *dnaK756 thr*::Tn*10*	C. Herman
SL590	594 *katE*::*lacZ*	C. Herman

SMR747	FC40 *recR252*::Tn*10-9*Kan	Rosenberg Laboratory Collection
SMR1982	FC40 *recG258*::Tn*10*miniKan	(Harris *et al.*, 1996)
SMR3180	SMR4562 *recJ*::Tn*10*Str	(Harris, 1997)
SMR3856	FC40 Lac$^+$ day 5 isolate	(McKenzie *et al.*, 1998)
SMR3858	FC40 Lac$^+$ day 5 isolate	(McKenzie *et al.*, 1998)
SMR3859	FC40 Lac$^+$ day 5 isolate	(McKenzie *et al.*, 1998)
SMR4562	Independent construction of FC40	(McKenzie *et al.*, 2000)
SMR4615	MG1655 *lacI*^{q} *lacI33*Ω*lacZ* Rif^R	(Ponder, 2006)
SMR4874	MG1655 Rif^R *lacI*^{q} *lacI33*Ω*lacZ* [F$^+$]	(Ponder, 2006)
SMR5075	SMR4562 Δ*recBCD*::P$_{lac}$ *red*$^+$ Kan	(Gumbiner-Russo *et al.*, 2001)
SMR5236	SMR4562 *rpoE2072*::Tn*10*dCam	This study
SMR5400	SMR4562 *sulA211 lexA51*(Def) Δ*psiB*::*cat*	(McKenzie *et al.*, 2000)
SMR6263	MG1655 *leu*::Tn*10*	(Ponder *et al.*, 2005)
SMR5833	SMR4562 [pKD46]	Rosenberg Laboratory Collection
SMR6276	SMR4562 Δ*araBAD567* Δ*attλ*::P$_{BAD}$*I-SceI*	(Ponder *et al.*, 2005)
SMR6280	SMR4562 Δ*araBAD567* Δ*attλ*::P$_{BAD}$*I-SceI* [F' *mhpA32*::miniTn*7*Kan(I-SceI site)]	(Ponder *et al.*, 2005)
SMR7037	SMR4562 *trpC3117*::Tn*10*Kan	(Lombardo *et al.*, 2004)
SMR8838	SMR4562 Day 5 Lac$^+$ isolate *rpoE2072*::Tn*10*dCam	SMR3856 x P1 (SMR5236)
SMR8839	SMR4562 Day 5 Lac$^+$ isolate *rpoE2072*::Tn*10*dCam	SMR3858 x P1 (SMR5236)
SMR8840	SMR4562 Day 5 Lac$^+$ isolate *rpoE2072*::Tn*10*dCam	SMR3859 x P1 (SMR5236)
SMR8842	CAG45114 *rpoE2072*::Tn*10*dCam	CAG45114 x P1 (SMR5236)
SMR8843	SMR8842 [pBA166]	SMR8842 x pBA166
SMR8844	SMR8842 [pTrc99a]	SMR8844 x pTrc99a
SMR8845	CAG45114 [pTrc99a]	CAG45114 x pTrc99a
SMR8846	CAG45114 [pBA166]	CAG45114 x pBA166
SMR8860	SMR4562 *dnaK756 thr*::Tn*10*	SMR4562 x P1 (SL471)
SMR8861	SMR4562 *dnaK756 thr*::Tn*10* *rpoE2072*::Tn*10*dCam	SMR8860 x P1 (SMR5236)
SMR8862	SMR4562 *dnaK756*	SMR8860 x P1 (MG1655)
SMR8863	SMR4562 *dnaK756 rpoE2072*::Tn*10*dCam	SMR8862 x P1 (SMR5236)
SMR8919	SL590 *rpoE2072*::Tn*10*dCam	SL590 x P1 (SMR5236)
SMR9182	MG1655 Rif^R *lacI*^{q} *lacI33*Ω*lacZ* *rpoE2072*::Tn*10*dCam	SMR4615 x P1 (SMR5236)

SMR9183	MG1655 Rif^R *lacI*^{q} *lacI33ΩlacZ* *rpoE2072*::Tn*10*dCam[F^+]	SMR4874 x P1 (SMR5236)
SMR9186	SMR4562 *recR*::Tn*10-9*Kan *recJ*::Tn*10*Str	SMR747 x P1 (SMR3180)
SMR9191	SMR6276 *rpoE2072*::Tn*10*dCam	SMR6276 x P1 (SMR5236)
SMR10168	SMR6280 *rpoE2072*::Tn*10*dCam	SMR6280 x P1 (SMR5236)
SMR10266	SMR4562 Δ*ydcQ*::FRTKanFRT	SMR4562 x P1 (JW1433)
SMR10267	SMR10266 *rpoE2072*::Tn*10*dCam	SMR10266 x P1(SMR5236)
SMR10268	SMR4562 *rpoE*::ΩCm	SMR4562 x P1(CAG22216)
SMR10277	SMR10266 *rpoE*::ΩCm	SMR10266 x P1 (CAG22216)
SMR10308	SMR4562 [F' *mbhA*::FRT*cat*FRT *dinB*O^{c1}]	(Galhardo *et al.*, 2009)
SMR10309	SMR4562 [F' *mbhA*::FRT*cat*FRT *dinB*O^{c2}]	(Galhardo *et al.*, 2009)
SMR10368	SMR4562 Δ*psiB*::FRTKanFRT [pKD46]	Short homology into SMR5833 with FRTKanFRT
SMR10369	SMR4562 *sulA211 lexA51*(Def) Δ*psiB*::FRTKanFRT	SMR5400 x P1(SMR10368)
SMR10370	SMR10369 *rpoE2072*::Tn*10*dCam	SMR10369 x P1(SMR5236)
SMR10464	SMR4562 [F'*mbhA*::FRT *dinB*O^{c1}]	pCP20 x SMR10308
SMR10465	SMR4562 [F'*mbhA*::FRT *dinB*O^{c2}]	pCP20 x SMR10309
SMR10466	SMR4562 *rpoE2072*::Tn*10*dCam [F'*mbhA*::FRT *dinB*O^{c1}]	SMR10464 x P1(SMR5236)
SMR10467	SMR4562 *rpoE2072*::Tn*10*dCam [F'*mbhA*::FRT *dinB*O^{c2}]	SMR10465 x P1(SMR5236)
SMR10472	SMR4562 [pPdinB]	SMR4562 x pPdinB
SMR10474	SMR10369 [pPdinB]	SMR10369 x pPdinB
SMR10475	SMR10370 [pPdinB]	SMR10370 x pPdinB
SMR10479	SMR5236 [pPdinB]	SMR5236 x pPdinB
SMR10658	SMR4562 Δ*recBCD*::*red*$^+$ Kan	SMR4562 x P1 (SMR5075)
SMR10659	SMR10658 *rpoE2072*::Tn*10*dCam	SMR10658 x P1 (SMR5236)
SMR11033	FC40 Lac^+ mutant with secondary mutation: temperature-sensitive for growth on minimal medium at 42°C	(Torkelson *et al.*, 1997)
SMR11034	FC40 Lac^+ mutant with secondary mutation: temperature-sensitive for growth on minimal medium at 42°C	(Torkelson *et al.*, 1997)
SMR11035	FC40 Lac^+ mutant with secondary mutation:: *upp* (5-fluorouracil resistant)	(Torkelson *et al.*, 1997)
SMR11036	FC40 Lac^+ mutant with secondary mutation: Mal^-	(Torkelson *et al.*, 1997)
SMR11037	FC40 Lac^+ mutant with secondary mutation: Xyl^-	(Torkelson *et al.*, 1997)
SMR11038	SMR11033 *rpoE2072*::Tn*10*dCam	SMR11033 x P1 (SMR5236)
SMR11039	SMR11034 *rpoE2072*::Tn*10*dCam	SMR11034 x P1 (SMR5236)
SMR11040	SMR11035 *rpoE2072*::Tn*10*dCam	SMR11035 x P1 (SMR5236)

SMR11041	SMR11036 *rpoE2072*::Tn*10*dCam	SMR11036 x P1(SMR5236)
SMR11042	SMR11037 *rpoE2072*::Tn*10*dCam	SMR11037 x P1(SMR5236)
SMR11044	*yfhH*::FRTKanFRT *rpoE2072*::Tn*10*dCam	JW2545 x P1 (SMR5236)
Plasmids		
pBA166	pTrc99a Amp^R, IPTG-inducible YYF peptide	(Walsh *et al*., 2003)
pCP20	Yeast Flp recombinase on a temperature-sensitive replicon λcI*ts857*	(Datsenko & Wanner, 2000)
pKD4	Source of FRTKanFRT	(Datsenko & Wanner, 2000)
pKD46	*ori101 repA101*ts P_{BAD}-*gam-bet-exo* Amp^R	(Datsenko & Wanner, 2000)
pPdinB	P_{dinB}::*lacZ*	(Galhardo *et al*., 2009)
pTrc99a	pBR322 ori, Amp^R	Amersham Pharmacia Biotech

Supplementary Figures

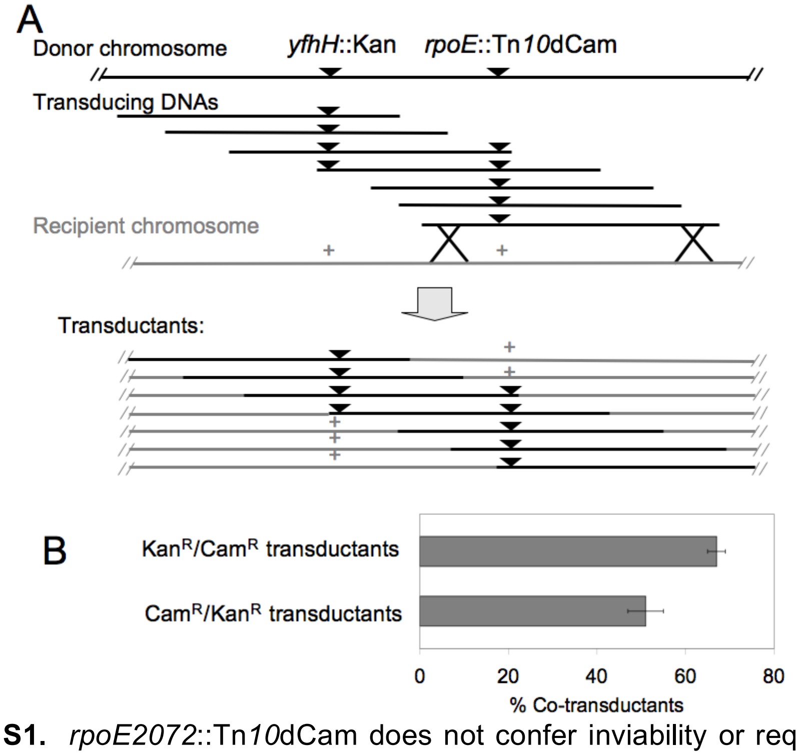

Fig. S1. *rpoE2072*::Tn*10*dCam does not confer inviability or require acquisition of suppressor mutations for cell viability.

A. Co-transduction strategy to determine whether *rpoE2072*::Tn*10*dCam impairs the *rpoE* essential function. Black lines represent transducing DNA from P1 phage grown on donor strain SMR11044 carrying *rpoE2072*::Tn*10*dCam mutation and linked kanamycin resistance (Kan^R) marker in *yfhH*. Gray lines indicate the recipient chromosome of the $rpoE^+$ $yfhH^+$ strain. The frequency of Kan^R Cam^R co-transductants reflects the distance between the two antibiotic-resistance markers. Selection on chloramphenicol and screening for Kan^R will give a co-transductant frequency equivalent to selection on kanamycin and screening for chloramphenicol

resistance (Cam^R) only if neither gene is essential for growth. If the *rpoE* mutation caused lethality unless accompanied by an unlinked suppressor mutation, then a low frequency of Cam^R would be expected among Kan^R colonies because only those recipient cells carrying a rare spontaneous suppressor mutation that allowed growth with the otherwise lethal *rpoE* mutation would produce a viable Cam^R co-transductant.

B. Co-transduction of *rpoE*::Tn*10d*Cam with *yfhH*::FRTKan shows little bias against the *rpoE*::Tn*10d*Cam mutation. Percent co-transductants represent mean ± range from 2 independent experiments. *rpoE*$^+$ *yfhH*$^+$ recipient strain SMR4562 was transduced with P1 grown on *rpoE2072*::Tn*10*dCam *yfhH*::FRTkan donor strain SMR11044. Transductants were selected on either LBH-kanamycin then screened for chloramphenicol resistance (Cam^R / Kan^R data), or LBH-chloramphenicol, then screened for kanamycin resistance (Kan^R / Cam^R data).

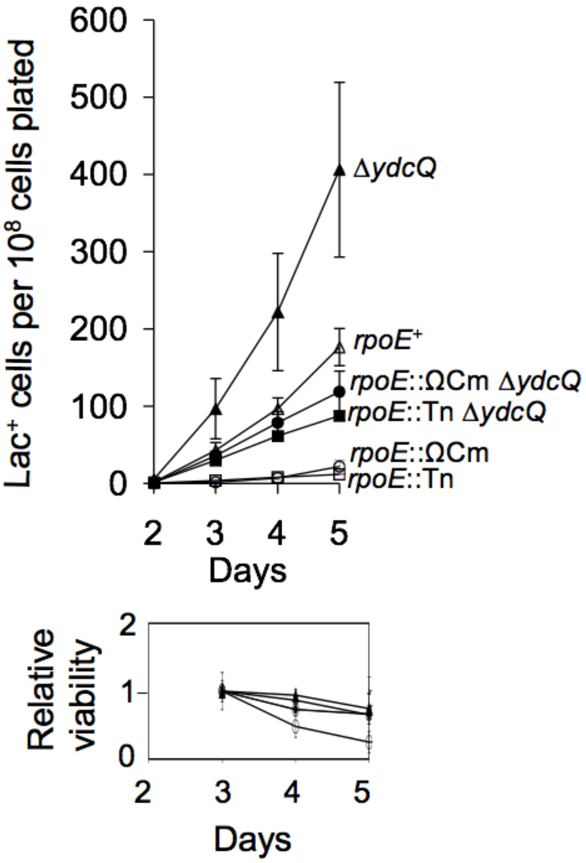

Fig. S2. *rpoE* mutations decrease Lac$^+$ stress-induced mutation. Strains are *rpoE*$^+$, SMR4562 (△); *rpoE2072*::Tn*10*dCam, SMR5236 (□); *rpoE*ΩCm; SMR10268 (○); Δ*ydcQ*, SMR10266 (▲); *rpoE*::Tn*10*dCam Δ*ydcQ*, SMR10267 (■); *rpoE*ΩCm Δ*ydcQ*, SMR10277 (●). (A) Assay performed at 30°. Values are means ± one SEM for at least three independent cultures of each strain in one experiment. Where not visible, error bars are smaller than the symbol. A second experiment yielded similar results. (B) Relative viability of the Lac$^-$ population was monitored as in Fig. 3.

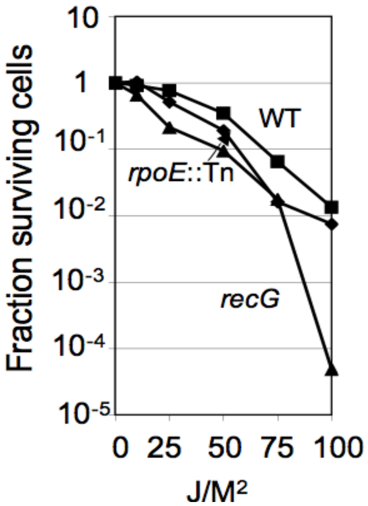

Fig. S3. *rpoE2072*::Tn*10*dCam mutation does not confer UV sensitivity. Appropriate dilutions of overnight cultures grown on LBH medium were plated on LBH medium and exposed to different doses of UV irradiation. Wild-type, SMR4562 (■); *rpoE2072*::Tn*10*dCam, SMR5236 (◆); *recG*, SMR1982 (▲).

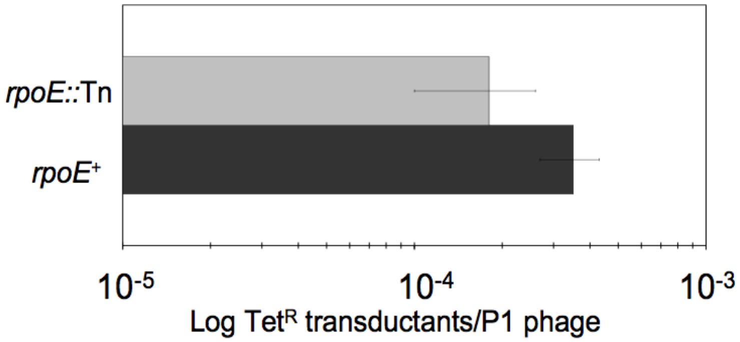

Fig. S4. *rpoE2072*::Tn*10*dCam cells are proficient at transductional recombination. Phage P1 grown on strain SMR6263 was used to transduce *leu*::Tn*10* into recipient strains *rpoE*$^+$, SMR4615, and *rpoE2072*::Tn*10*dCam, SMR9182. Transductant frequencies are means from three P1 transductions with recipient in cells in excess (MOIs of <0.01 phage per recipient cell).

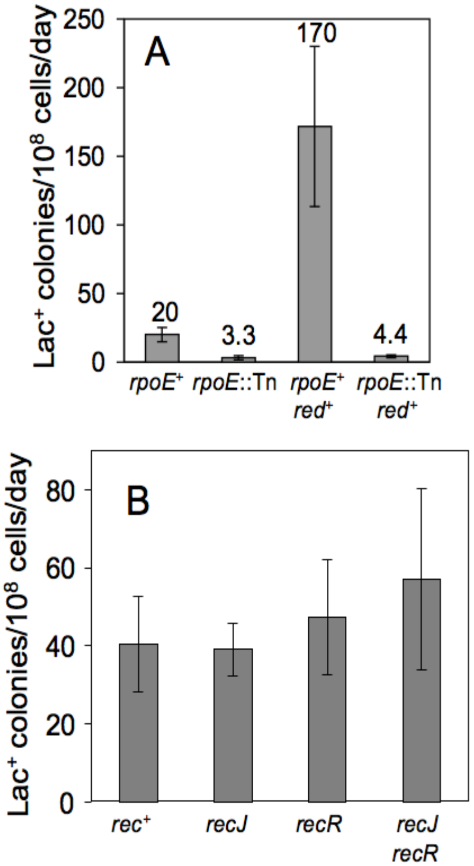

Fig. S5. Regulation of RecBCD, RecJ, or RecR expression is not the role of the σ^E response in stress-induced mutagenesis. Stress-induced mutation rates (Lac$^+$ cfu per 10^8 cells per day) were calculated from colonies appearing from days 3-5 and represent the average of 3 experiments ± SEM. Experiments were performed at 37° per Experimental Procedures. Viability of all cultures was monitored as described by Harris *et al.* (1996). (A) Substitution of the phage λ Red recombination functions for *E. coli* RecBCD does not alleviate the requirement for RpoE$^+$ function in stress-induced mutagenesis. Strains: *rpoE*$^+$, SMR4562; *rpoE*::Tn, SMR5236; *rpoE*$^+$ *red*$^+$, SMR10658; *rpoE*::Tn *red*$^+$, SMR10659, in which *red*$^+$ strains carry the phage λ *red* recombination genes and are deleted for *recBCD.* (B) Neither RecR nor simultaneous RecR and RecJ loss impair stress-induced mutagenesis. Strains: *rec*$^+$, SMR4562; *recJ*, SMR3180; *recR*, SMR747; *recJ recR* SMR9186.

Supplementary Experimental Procedures

Screen for mutants defective in stress-induced Lac reversion

Lac assay strain FC40 (Table S5) was mutagenized with the 1.3 kb transposon Tn*10*dCam using lambda NK1324 (Kleckner *et al.*, 1991) selecting for chloramphenicol resistant (Cam^R) isolates on M9 medium with 0.1% lactose, 0.025% glycerol and chloramphenicol. Plates were incubated at 38-40°C (to prevent lysogeny) for 48 hrs and then at 37°C for 24 additional hrs to allow larger colonies to form. The colonies were then replica plated to M9 medium with 0.1% lactose, chloramphenicol and X-gal, and to M9 medium with 0.1% glycerol and chloramphenicol at 37°C. Potential mutants defective in stress-induced mutation were identified as those that yielded few blue Lac$^+$ mutant papillae (small colonies) in the patches on M9 lactose after 6-7 days of incubation relative to wild-type control patches. 427 possible mutants were identified among approximately 40,000 screened and were purified from the M9 glycerol replica plate. Of these, approximately 130 were confirmed to be deficient in a qualitative spot test for reversion to Lac$^+$ in which 5 μl of a saturated M9 glycerol culture were spread to a quarter of an M9 lactose plate and Lac$^+$ colonies were scored each day for several days. Each of these 130 transposons was moved *via* phage P1-mediated transduction (Miller, 1992) into FC40 selecting Cam^R and the stress-induced-mutation-defective phenotype of two purified transductants from each cross was rescored in spot tests. 106 insertions had phenotypes and the transductants were used in all further studies.

To identify chromosomal insertions and exclude further study of insertions causing F-

transfer deficiency, which are deficient for stress-induced mutation (Foster & Trimarchi, 1995, Galitski & Roth, 1995), the 106 insertion strains were used as donors in single culture quantitative mating experiments with the recipient SMR828, a derivative of P90C (Coulondre & Miller, 1977) carrying *zaj3053*::Tn*10* (Singer *et al.*, 1989a). Pro^{+} Tet^{R} transconjugants were selected and screened by replica plating for Cam^{R} (Cam^{R} indicates that the insertion was into the F'). 27 insertions were identified as being chromosomal and not having strong effects on F transfer by this test and were characterized by sequence analysis. Tn*10*dCam and flanking DNA were cloned as *Hin*dIII or *Aat*II fragments (neither cuts in the Tn*10*dCam) from genomic DNA preparations of each mutant into *Hin*dIII or *Aat*II digested pBR322 selecting for Cam^{R} transformants in *E. coli* DH5α (Grant *et al.*, 1990). Insertion sites were determined by sequencing outward from the Tn*10*dCam with an outward reading primer (5'GGTGGTGCGTAACGGCAAAAG). BLAST analysis (Altschul *et al.*, 1990) was used to identify the disrupted genes.

References

Ades, S. E., I. L. Grigorova & C. A. Gross, (2003) Regulation of the alternative sigma factor sigma(E) during initiation, adaptation, and shutoff of the extracytoplasmic heat shock response in Escherichia coli. *J Bacteriol* **185**: 2512-2519.

Altschul, S. F., W. Gish, W. Miller, E. W. Myers & D. J. Lipman, (1990) Basic local alignment search tool. *J Mol Biol* **215**: 403-410.

Baba, T., T. Ara, M. Hasegawa, Y. Takai, Y. Okumura, M. Baba, K. A. Datsenko, M. Tomita, B. L. Wanner & H. Mori, (2006) Construction of Escherichia coli K-12 in-frame, single-gene knockout mutants: the Keio collection. *Mol Syst Biol* **2**: 2006 0008.

Cairns, J. & P. L. Foster, (1991) Adaptive reversion of a frameshift mutation in Escherichia coli. *Genetics* **128**: 695-701.

Coulondre, C. & J. H. Miller, (1977) Genetic studies of the lac repressor. IV. Mutagenic specificity in the lacI gene of Escherichia coli. *J Mol Biol* **117**: 577-606.

Datsenko, K. A. & B. L. Wanner, (2000) One-step inactivation of chromosomal genes in *Escherichia coli* K-12 using PCR products. *Proc Natl Acad Sci U S A* **97**: 6640-6645.

Foster, P. L. & J. M. Trimarchi, (1995) Adaptive reversion of an episomal frameshift mutation in *Escherichia coli* requires conjugal functions but not actual conjugation. *Proc Natl Acad Sci U S A* **92**: 5487-5490.

Galhardo, R. S., R. Do, M. Yamada, E. C. Friedberg, P. J. Hastings, T. Nohmi & S. M. Rosenberg, (2009) DinB upregulation is the sole role of the SOS response in stress-induced mutagenesis in Escherichia coli. *Genetics* **182**: 55-68.

Galitski, T. & J. R. Roth, (1995) Evidence that F' transfer replication underlies apparent adaptive mutation. *Science* **268**: 421-423.

Grant, S. G. N., J. Jesse, F. R. Bloom & D. Hanahan, (1990) Differential plasmid rescue from transgenic mouse DNAs into *Escherichia coli* methylation-restriction mutants. *Proc. Natl. Acad. Sci. (USA)* **87**: 4645-4649.

Gumbiner-Russo, L. M., M. J. Lombardo, R. G. Ponder & S. M. Rosenberg, (2001) The TGV transgenic vectors for single-copy gene expression from the Escherichia coli chromosome. *Gene* **273**: 97-104.

Harris, R. S., (1997) *On a Molecular Mechanism of Adaptive Mutation in* E. coli. Ph.D. thesis, University of Alberta, Department of Biological Sciences.

Harris, R. S., K. J. Ross & S. M. Rosenberg, (1996) Opposing roles of the holliday junction processing systems of Escherichia coli in recombination-dependent adaptive mutation. *Genetics* **142**: 681-691.

Hastings, P. J., H. J. Bull, J. R. Klump & S. M. Rosenberg, (2000) Adaptive amplification: an inducible chromosomal instability mechanism. *Cell* **103**: 723-731.

Kleckner, N., J. Bender & S. Gottesman, (1991) Uses of transposons with emphasis on Tn*10*. *Methods Enzymol.* **204**: 140-180.

Lombardo, M. J., I. Aponyi & S. M. Rosenberg, (2004) General stress response regulator RpoS in adaptive mutation and amplification in Escherichia coli. *Genetics* **166**: 669-680.

McKenzie, G. J., R. S. Harris, P. L. Lee & S. M. Rosenberg, (2000) The SOS response regulates adaptive mutation. *Proc Natl Acad Sci U S A* **97**: 6646-6651.

McKenzie, G. J., M. J. Lombardo & S. M. Rosenberg, (1998) Recombination-dependent mutation in Escherichia coli occurs in stationary phase. *Genetics* **149**: 1163-1165.

Miller, J. H., (1992) *A Short Course in Bacterial Genetics*. Cold Spring Harbor Laboratory Press, Cold Spring Harbor, N. Y.

Ponder, R. G., (2006) *Error-Prone DNA Double-Strand Break Repair In Stress-Induced Mutation.* Ph.D. thesis, Baylor College of Medicine, Department of Molecular and Human Genetics.

Ponder, R. G., N. C. Fonville & S. M. Rosenberg, (2005) A switch from high-fidelity to error-prone DNA double-strand break repair underlies stress-induced mutation. *Mol Cell* **19**: 791-804.

Rouviere, P. E., A. De Las Penas, J. Mecsas, C. Z. Lu, K. E. Rudd & C. A. Gross, (1995) rpoE, the gene encoding the second heat-shock sigma factor, sigma E, in Escherichia coli. *Embo J* **14**: 1032-1042.

Singer, M., T. A. Baker, G. Schnitzler, S. M. Deischel, M. Goel, W. Dove, K. J. Jaacks, A. D. Grossman, J. W. Erickson & C. A. Gross, (1989a) A collection of strains containing genetically linked alternating antibiotic resistance elements for genetic mapping of *Escherichia coli*. *Microbiology Reviews* **53**: 1-24.

Singer, M., T. A. Baker, G. Schnitzler, S. M. Deischel, M. D. Goel, W., K. J. Jaacks, A. D. Grossman, J. W. Erickson & C. A. Gross, (1989b) A collection of strains containing genetically linked alternating antibiotic resistance elements for genetic mapping of Escherichia coli. *Microbiological Reviews* **53**: 1-24.

Torkelson, J., R. S. Harris, M. J. Lombardo, J. Nagendran, C. Thulin & S. M. Rosenberg, (1997) Genome-wide hypermutation in a subpopulation of stationary-phase cells underlies recombination-dependent adaptive mutation. *Embo J* **16**: 3303-3311.

Walsh, N. P., B. M. Alba, B. Bose, C. A. Gross & R. T. Sauer, (2003) OMP peptide signals initiate the envelope-stress response by activating DegS protease via relief of inhibition mediated by its PDZ domain. *Cell* **113**: 61-71.

Wanner, B. L., (1986) Novel regulatory mutants of the phosphate regulon in Escherichia coli K-12. *J Mol Biol* **191**: 39-58.
